# RNA-binding proteins regulating the CD44 alternative splicing

**DOI:** 10.3389/fmolb.2023.1326148

**Published:** 2023-12-01

**Authors:** Diana Maltseva, Alexander Tonevitsky

**Affiliations:** ^1^ Faculty of Biology and Biotechnology, HSE University, Moscow, Russia; ^2^ Shemyakin-Ovchinnikov Institute of Bioorganic Chemistry, Russian Academy of Sciences, Moscow, Russia

**Keywords:** CD44 alternative splicing regulation, CD44 isoform, RNA-binding protein, cancer, ESRP1

## Abstract

Alternative splicing is often deregulated in cancer, and cancer-specific isoform switches are part of the oncogenic transformation of cells. Accumulating evidence indicates that isoforms of the multifunctional cell-surface glycoprotein CD44 play different roles in cancer cells as compared to normal cells. In particular, the shift of CD44 isoforms is required for epithelial to mesenchymal transition (EMT) and is crucial for the maintenance of pluripotency in normal human cells and the acquisition of cancer stem cells phenotype for malignant cells. The growing and seemingly promising use of splicing inhibitors for treating cancer and other pathologies gives hope for the prospect of using such an approach to regulate CD44 alternative splicing. This review integrates current knowledge about regulating CD44 alternative splicing by RNA-binding proteins.

## 1 Introduction

CD44 is a multifunctional transmembrane glycoprotein that is widely expressed and plays an essential role in physiological activities in normal cells throughout the body. CD44 was first discovered to be present on the cell membrane of haematopoietic cells (Jalkanen et al., 1986) and after that, its expression was noted in different non-haematopoietic cells (Fox et al., 1994). The first studies of the physiological role of CD44 showed that CD44-deficient mice are viable without obvious developmental defects and show no overt abnormalities as adults. However, during development, they had impaired lymphocyte trafficking into the thymus (Protin et al., 1999). During further decades CD44 emerged as a regulator of malignant progression and metastasis formation due to its involvement in cell proliferation, adhesion, cytoskeleton rearrangement, migration, angiogenesis, inflammation, metabolism (regulating glucose and lipid homeostasis) (Zöller, 2011; Senbanjo and Chellaiah, 2017; Chaffer and Goetz, 2018; Chen et al., 2020; Guo Q. et al., 2022; Weng et al., 2022). Notably, CD44 is a commonly accepted marker of cancer stem cells (CSC) of different cancer entities including breast, colon, gastric, pancreas, glioma, ovarian (Zöller, 2011; Hu and Fu, 2012; Yan et al., 2015; Skandalis et al., 2019), and of epithelial to mesenchymal transition (EMT) a process vital for distant metastasis formation (Cho et al., 2012; Zhang et al., 2012; Jiang et al., 2015).

The marked multifunctionality and variability of the CD44 protein are ensured by the existence of its multiple forms, which mainly originate in alternative splicing and are further amplified by extensive and often isoform-specific posttranslational modifications including *N*- and *O*-glycosylation, phosphorylation, and glycosaminoglycan attachment (Fox et al., 1994; Ponta et al., 2003; Zöller, 2011; Wang et al., 2018). Thus, CD44 is a family of transmembrane glycoproteins with a high heterogeneity in molecular weight (85–250 kDa). Alternative splicing (AS) is often deregulated in cancer, and cancer-specific isoform switches are part of the oncogenic transformation of cells (Di et al., 2018; Zhang et al., 2021; Shaw et al., 2022; Bradley and Anczuków, 2023). Indeed, accumulating evidence supports the concept that CD44 isoforms play different roles in cancer cells as compared to their normal counterparts (Zöller, 2011; Bhattacharya et al., 2018; Xu et al., 2020). In particular CD44 isoform switches have been shown during EMT and acquisition of CSC properties (Bhattacharya et al., 2018; Zhang et al., 2019). Moreover, the shift of CD44 isoforms is required for EMT (Reinke et al., 2012). The understanding of the mechanisms of alternative splicing and the occurrence of variant isoforms of CD44 is essential not only to a deeper insight into malignant progression but may also provide a new generation of splicing inhibitors as therapies for cancer (Bonnal et al., 2020; Rogalska et al., 2022). The major experimentally tested regulators of alternative splicing of CD44 in cancer have been described earlier by Prochazka and co-authors (Prochazka et al., 2014). Our review will focus on new data concerning RNA binding proteins, which were recently shown as an essential regulator in CD44 isoform switching.

## 2 Overview of CD44 isoforms

**FIGURE 1:**
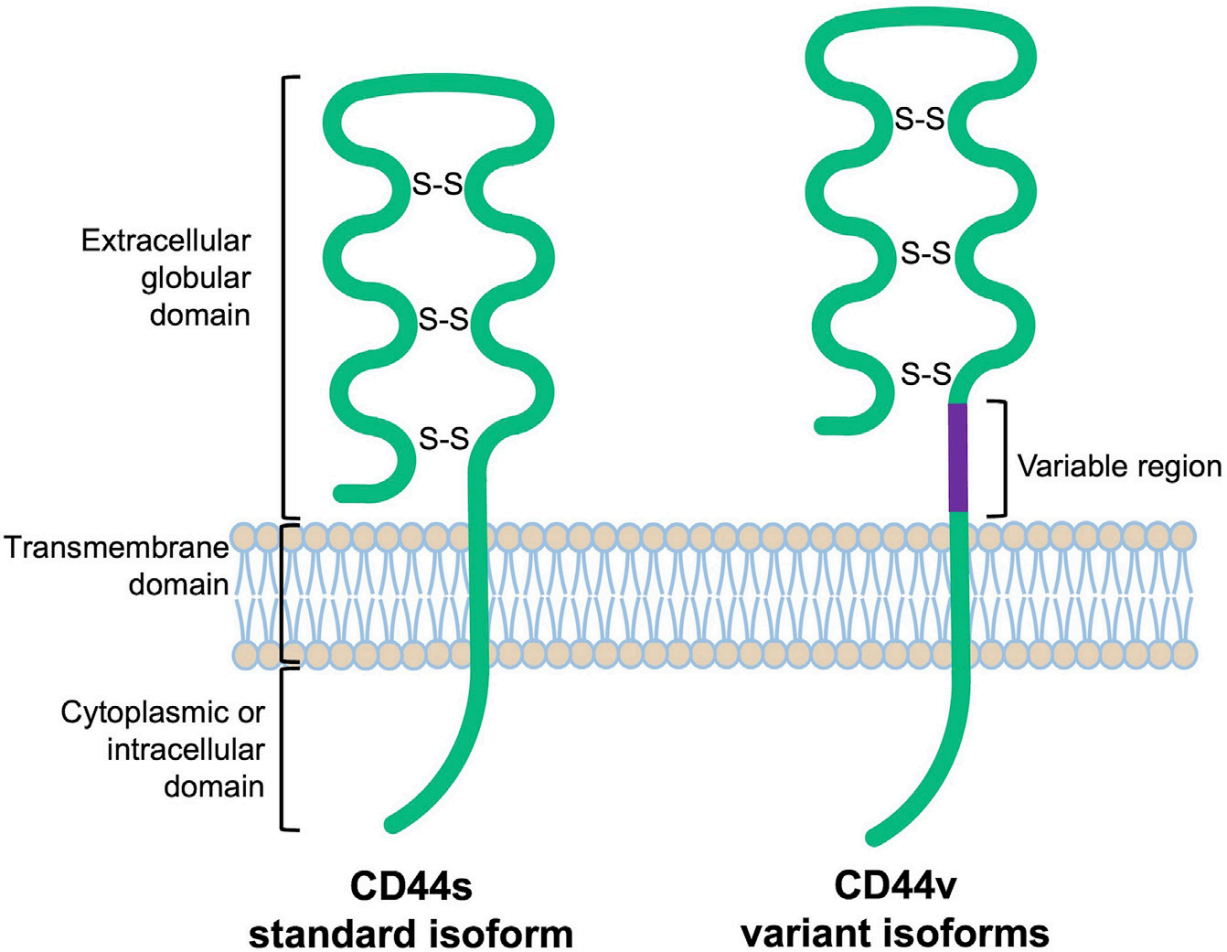
Schematic protein structure of CD44 molecules [extrapolated from (Naor et al., 1997)].

**FIGURE 2:**
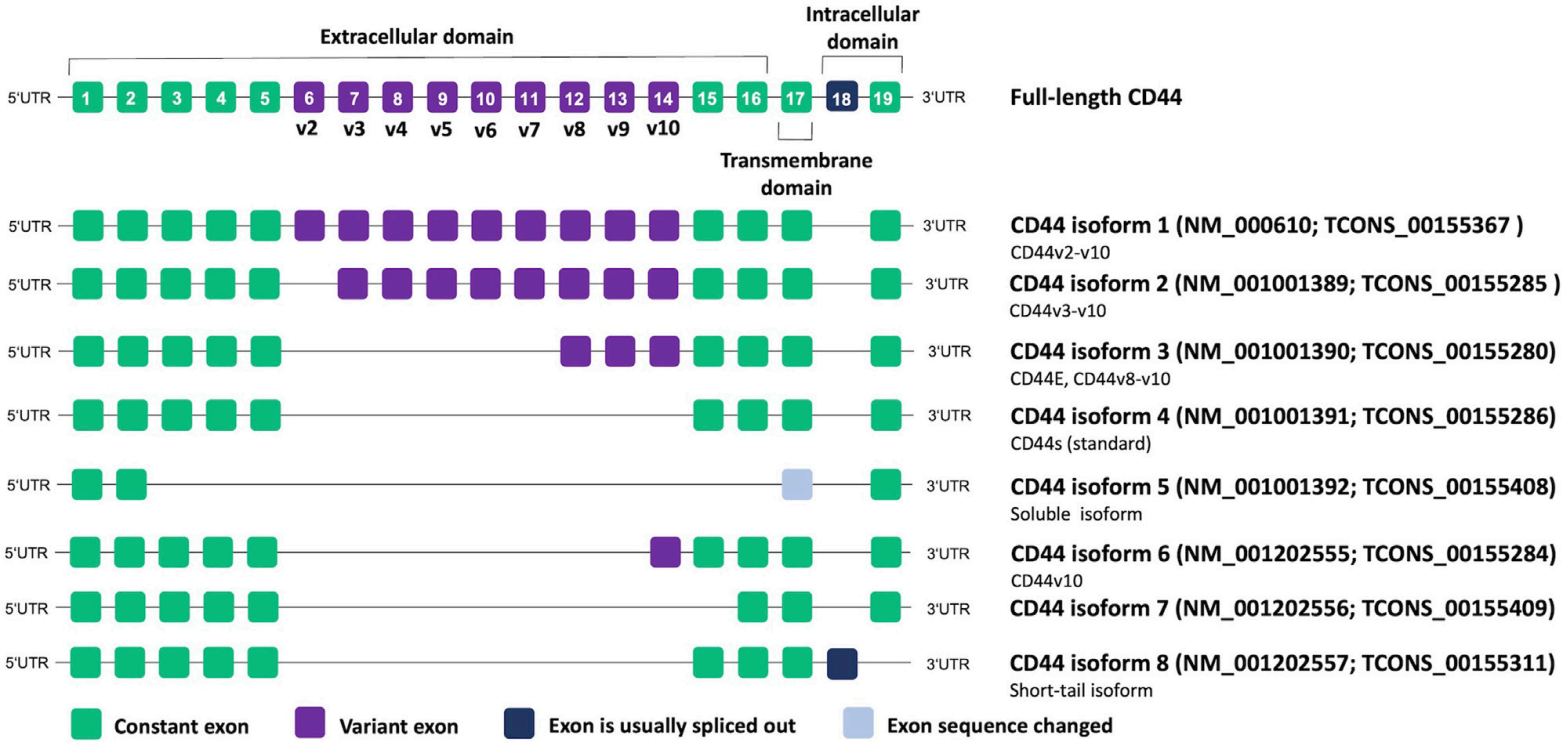
CD44 genomic organization and alternative splicing according to NCBI database. Transcript IDs are also provided according to the FLIBase database (Shi et al., 2023).

CD44 proteins have a common structure consisting of three major domains: an extracellular or ectodomain (ECD), a transmembrane domain (TMD) and a cytoplasmic or intracellular domain (ICD) (Figure 1) (Ponta et al., 2003; Zöller, 2011; Wang et al., 2018). The ECD comprises an N-terminal globular domain and a membrane-proximal region, which may include variant exons (variable region). All CD44 proteins are encoded by one single gene present on chromosome 11 in humans, which includes 19 exons so that alternative splicing gives rise to plentiful isoforms (Figure 2) (Screaton et al., 1992; Azevedo et al., 2018). According to NCBI database, eight CD44 isoforms are commonly accepted as biologically expressed (Figure 2), and the existence of 27 other isoforms was predicted. Nonetheless, it should be mentioned that some data exist that other CD44 isoforms except these eight do exist (Bánky et al., 2012; Marzese et al., 2015; Kim et al., 2018). In a recent study, full-length mRNA transcripts from diverse normal and cancerous human tissues have been profiled using long-read sequencing techniques (Shi et al., 2023). The RNA sequencing data were collected in the FLIBase repository. Based on the FLIBase data, more than two hundred CD44 isoforms were detected in human cells. The shortest or standard CD44 isoform (CD44s, isoform 4) contains only constant (invariant) exons (the first one to five and the last four 15–17 and 19). Exon 18 is mostly spliced out in humans. CD44s is ubiquitously expressed in most tissues. The ECD of this isoform is composed of only an N-terminal globular domain (Figure 1). Including variant exons v2-v10 (variant exon v1 is not present in humans) into a membrane-proximal region of the ECD gives larger isoforms which are expressed in only a few epithelial tissues, mainly in proliferating cells, and in cancer cells of several cancer entities as well. CD44 variant isoforms are often numbered depending on the inclusion of corresponding exons, e.g., CD44 isoform 1 contains CD44v2-v10, isoform 2 contains CD44v3-v10 and isoform 3 contains CD44v8-v10.

Importantly, authors of studies often adopt a nomenclature based on commercial names of the used monoclonal antibodies, highlighting the targeted variant exon, and disregard that the analysis of a specific variant exon can result in the detection of all isoforms containing it instead of **only** one particular protein. Thus, such isoforms as CD44v3, CD44v6, and CD44v9 started being the most associated with cancer (Azevedo et al., 2018). However, most of these studies dealt only with antibodies specific to the corresponding individual exons. Thus, the lack of nomenclature standardization makes it difficult to interpret the results presented in the articles and requires careful conclusions about which isoform/isoforms are actually in question. In our review, we prefer to use CD44 isoform designations according to NCBI nomenclature if possible.

CD44 proteins are primarily considered as cell adhesion molecules as they contain binding sites for hyaluronan (Underhill, 1992), collagen (Ishii et al., 1993), laminins (Ishii et al., 1993; Hibino et al., 2004), fibronectin (Jalkanen and Jalkanen, 1992), E-/P-/L-selectins (Hanley et al., 2006). However, due to their signaling functions, CD44 proteins play essential roles in intercellular communication and numerous other cellular functions associated with it (Ponta et al., 2003; Wang et al., 2018). CD44 variant isoforms encode additional peptides in the membrane-proximal region, which provide binding sites for other molecules including cytokines and growth factors. This configuration allows CD44 transmembrane glycoproteins to emerge as a multidomain platform, which integrates various extracellular information. We will not dwell on the mechanisms of signaling in details, information for a deeper understanding can be found in these excellent reviews (Orian-Rousseau and Sleeman, 2014; Ouhtit et al., 2018; Wang et al., 2018; Mesrati et al., 2021; Guo Q. et al., 2022).

CD44 is a well-known normal intestinal stem cell marker (ISC) (Habowski et al., 2020), and its alternative splicing should be tightly controlled in the crypt-villus axis (Orian-Rousseau and Sleeman, 2014). Thus, ISCs residing at the crypt base in mice express mRNA encoding CD44 isoforms v4–v10, v6-v10, v7-v10 and isoform 3 (v8-v10), but do not express the standard isoform 4 (CD44s) (Zeilstra et al., 2013). Progenitor cells from a transit-amplifying compartment (daughter cells of ISCs) express mRNAs encoding CD44 isoforms containing v6-v10, v7-v10, isoform 3 (v8-10), as well as standard isoform 4 (CD44s). Interestingly, human ISCs display a somewhat different repertoire of CD44 isoforms than mice presenting CD44v6-v10, v7-v10, isoform 3 (v8-10), and standard isoform 4 (Zeilstra et al., 2013). However, neoplastic epithelial cells from microadenomas of familial adenomatous polyposis patients demonstrate an expression profile of CD44 mRNAs more similar to mice ISCs, suggesting involvement of variant isoforms at early stages of human CRC (Zeilstra et al., 2013). An earlier study reported that the lower part of the crypts express CD44 isoform containing exon v9 but not exons v4 and v6 in humans (Mackay et al., 1994).

## 3 CD44 isoforms in cancer

A huge amount of data indicates that different CD44 isoforms play a role in many types of cancer [reviewed in (Chen et al., 2018; Mesrati et al., 2021; Yaghobi et al., 2021)], and their cellular functions can both overlap and be distinct. Sometimes, the information about the functions of CD44 isoforms is controversial, which complicates our current understanding of their roles in malignancy and cancer progression (Table 1). Thus, elevated expression of CD44 isoform 3 occurs in breast (Yae et al., 2012), gastric (Ishimoto et al., 2011; Lau et al., 2014), bladder (Miyake et al., 2002), esophageal (Kagami et al., 2018), gallbladder cancer (Yamaguchi et al., 2000), thyroid (Kawai et al., 2019), ovarian (Sosulski et al., 2016), colorectal cancer (Boman et al., 2023; Everest-Dass et al., 2023), melanoma (Zhang et al., 2016) and leukemia as well (Holm et al., 2015). While in breast, esophageal, and gallbladder cancer CD44 isoform 3 is associated with a more metastatic phenotype and poor prognosis [e.g., (Yamaguchi et al., 2000; Yae et al., 2012; Kagami et al., 2018)], it does not do so in ovarian cancer (Sosulski et al., 2016). Sometimes, e.g., in the case of colorectal cancer, CD44 isoform 3 has been shown associated with both poor prognosis/higher recurrence rate (Yamaguchi et al., 2016) and good prognosis/lower recurrence rate (Mashita et al., 2014; Everest-Dass et al., 2023), pointing to the possible greater significance of the ratio of CD44 isoforms. Indeed, the high ratio of CD44 isoform 4/CD44 variant exon v9 in patients with colorectal cancer shows a significantly poorer prognosis than the low CD44 isoform 4/CD44 variant exon v9 ratio (Mashita et al., 2014). In prostate cancer, CD44 isoform 3 is associated with CSC features (Zeng et al., 2013). However, in breast (Zhang et al., 2019), pancreatic (Li et al., 2014) and ovarian (Bhattacharya et al., 2018) cancers CSC features are determined by CD44 isoform 4. CD44 isoform 4 has been showing to play a critical role in the mesenchymal phenotype of many cancers (Primeaux et al., 2022), including hepatocellular carcinoma cells (Mima et al., 2012), breast cancer cells (Brown et al., 2011; Preca et al., 2015), colorectal cancer cells (Mashita et al., 2014), ovarian cancer cells (Bhattacharya et al., 2018), and in EMT of CSCs of cutaneous squamous cell carcinoma (Biddle et al., 2013). Several studies showed that a switch in CD44 isoform expression from CD44v to CD44 standard isoform 3 is essential for EMT (Brown et al., 2011; Preca et al., 2015). All of the above-mentioned findings suggest that both CD44 isoform 3 and isoform 4 are expressed in cancer cells but play distinct roles in the different steps of cancer development. Thus, as it has been partially shown in colon cancer, CD44 isoform 4 can play an anti-tumor role during the initial malignant transformation but may later benefit metastasis formation (Everest-Dass et al., 2023). In gallbladder cancer CD44 isoform 4 is associated with a mesenchymal phenotype, increased chemotaxis, increased invasiveness, but lower tumorigenicity (Miwa et al., 2017). At the same time, the CD44 variant exon v9 expression is associated with an epithelial phenotype, decreased chemotaxis, decreased invasiveness, and unexpectedly increased tumorigenicity. In the review (Wang et al., 2018), one can find detailed information about the engagement of CD44 exons v6 and v3 in the maintenance of CSCs and tumor progression. It is plausible that regulation of CD44 splicing allows CSCs to maintain the hybrid E/M state correlated with higher stemness and tumorigenicity (Pradella et al., 2017). Thus, CD44 undergoes isoform switching in cancer cells (Primeaux et al., 2022) and understanding its regulation mechanisms is incredibly important for a deeper insight into malignant progression. Our review will focus on how tumor cells implement CD44 isoform switching. The main players here are certainly RNA-binding proteins as far as AS relies on them to recognize and bind target sequences in pre-mRNAs, which allows for the inclusion or skipping of alternative exons (Table 2).

**TABLE 1:** Roles of CD44 isoforms 3 and 4 in various malignant entities.

CD44 isoform	Biological functions	Malignant entity	References
CD44 isoform 3 (CD44v8-v10)	Overexpressed in tumor tissue	Bladder cancer	Miyake et al. (2002)
	Overexpressed in tumor tissue, Metastasis, Poor prognosis	Breast cancer	Yae et al. (2012)
	Overexpressed in tumor tissue	Colorectal cancer	Boman et al. (2023), Everest-Dass et al. (2023)
	Poor prognosis/higher recurrence rate	Colorectal cancer	Yamaguchi et al. (2016)
	Good prognosis/lower recurrence rate; The high ratio of CD44 isoform 4/isoform 3 (or variant exon v9) showed a significantly poorer prognosis than the low isoform 4/isoform 3 (or variant exon v9) ratio	Colorectal cancer	Mashita et al. (2014), Everest-Dass et al. (2023)
	Overexpressed in tumor tissue, Metastasis, Poor prognosis	Esophageal cancer	Kagami et al. (2018)
	Overexpressed in tumor tissue, Metastasis, Poor prognosis	Gallbladder cancer	Yamaguchi et al. (2000)
	Epithelial phenotype, Decreased chemotaxis, Decreased invasiveness, Unexpectedly increased tumorigenicity	Gallbladder cancer	Miwa et al. (2017)
	Overexpressed in tumor tissue	Gastric cancer	Ishimoto et al. (2011); Lau et al. (2014)
	Overexpressed in tumor tissue	Leukemia	Holm et al. (2015)
	Overexpressed in tumor tissue	Melanoma	Zhang et al. (2016)
	Overexpressed in tumor tissue, Presence of transmembrane CD44 isoform 3 on the surface of primary tumor cells was a marker of a highly epithelial tumor with better prognosis	Ovarian cancer	Sosulski et al. (2016)
	CSC features	Prostate cancer	Zeng et al. (2013)
	Overexpressed in tumor tissue	Thyroid cancer	Kawai et al. (2019)
CD44 isoform 4 (CD44s)	CSC features	Breast cancer	Zhang et al. (2019)
	Mesenchymal phenotype	Breast cancer	Brown et al. (2011), Preca et al. (2015)
	Mesenchymal phenotype	Colorectal cancer	Mashita et al. (2014)
	EMT of CSCs	Cutaneous squamous cell carcinoma	Biddle et al. (2013)
	Mesenchymal phenotype, Increased chemotaxis, Increased invasiveness, Unexpectedly lower tumorigenicity	Gallbladder cancer	Miwa et al. (2017)
	Mesenchymal phenotype	Hepatocellular carcinoma	Mima et al. (2012)
	CSC features	Ovarian cancer	Bhattacharya et al. (2018)
	Mesenchymal phenotype		
	CSC features	Pancreatic cancer	Li et al. (2014)

**TABLE 2:** RNA-binding proteins regulating CD44 variant exon splicing.

Protein	Effect on CD44 isoform expression	Other outcomes	Cancer type, cell line	References
AGGF1	• Promotes the inclusion of exons v4 and v5 (but not v8-v10 or v10) in *CD44* mRNA and decreases the level of CD44 isoform 4 in cells	Co-overexpression of AGGF1 with NONO or SFPQ, or DHX15 enhanced the inclusion of exons v4 and v5 in the CD44 minigene splicing reporter system	Human cervical carcinoma HeLa cell line	Zhao et al. (2022)
	• Promotes the inclusion of exons v4 and v5 in the CD44 minigene splicing reporter system			
AKAP8	Promotes the inclusion of exon v8 in the CD44 minigene splicing reporter system in AKAP8 dose-dependent manner	hnRNPM knockdown showed a moderate but insignificant increase in the AKAP8’s splicing activity	Human embryonic kidney cell line HEK293FT.	Hu et al. (2020)
CELF1	CELF1 knockdown reduced the inclusion of variable exons v7-v10 into mature *CD44* mRNAs	Simultaneous depletion of CELF1 and ELAVL1 reduced the inclusion of exons v7-v10 into mature *CD44* mRNAs even more than each protein alone	Human cervical carcinoma HeLa cell line	David et al. (2022)
DHX15	Promotes the inclusion of exons v4 and v5 in the CD44 minigene splicing reporter system		Human cervical carcinoma HeLa cell line	Zhao et al. (2022)
ELAVL1	ELAVL1 knockdown reduced the inclusion of variable exons v7-v10 into mature *CD44* mRNAs	Simultaneous depletion of CELF1 and ELAVL1 reduced the inclusion of exons v7-v10 into mature *CD44* mRNAs even more than each protein alone	Human cervical carcinoma HeLa cell line	David et al. (2022)
ESRP1	Promotes the expression of variant CD44 isoforms (isoform 3, isoforms containing exons v6-v10), switching from CD44 isoform 4 to variant isoforms	The incidence and extent of lung metastasis were reduced after orthotopic injection of mouse tumor cells into mouse mammary glands	Human breast cancer cell lines MDA-MB-231 and MCF7; mouse breast cancer cell line 4T1	Warzecha et al. (2009a)
				Yae et al. (2012)
				Preca et al. (2015)
	Promotes the expression of variant CD44 isoforms, switching from CD44 isoform 4 to variant isoforms	• The isoform switch to CD44 isoform 4 was required for the formation of breast tumors in mice	Human mammary epithelial cell line HMLE.	Brown et al. (2011)
		• CD44 isoform 4 activated Akt signaling		
		• ESRP1 knockdown enhanced mammosphere-forming ability in response to TGFβ treatment		Zhang et al. (2019)
		• CD44 isoform 4 activated the PDGFRβ/Stat3 cascade to promote CSC traits		
		• Inhibition of the CSC gene signature		
	Promotes the expression of variant CD44 isoforms, switching from CD44 isoform 4 to variant isoforms		Human pancreatic adenocarcinoma BxPC-3 cells	Preca et al. (2015)
	Switching from CD44 isoform 4 to variant isoforms	• ESRP1 knockdown increased migration and invasion	Human epithelial ovarian cancer cell lines HO8910 and SKOV3	Chen et al. (2017)
		• Overall switching from mesenchymal to epithelial phenotype of cells		Jeong et al. (2017)
	ESRP1 knockdown promoted an upregulation of CD44 isoform 4 and downregulation of the CD44 variant isoforms		Human colorectal cancer cell line HCT-116	Vadlamudi and Kang (2022)
	Promotes conversion from CD44v9-v10 to CD44v7-v10		Human fully differentiated human foreskin fibroblasts	Kim et al. (2018)
	ESRP1 knockdown downregulated CD44v7-v10 expression and upregulated of CD44v9-v10		Undifferentiated H9 human embryonic stem cell	Kim et al. (2018)
	ESRP1 knockdown stimulated switching from the CD44 variant isoforms to the CD44 isoform 4	ESRP1 knockdown enhanced cell motility	Human head and neck squamous cell carcinoma cell lines SAS and HSC4	Ishii et al. (2014)
	ESRP1 knockdown decreased the expression of CD44 isoforms containing exon v6	ESRP1 knockdown significantly reduced the migration of cells under HGF treatment	Human cell lines MB and LH derived from melanoma lymph node metastases	Marzese et al. (2015)
	ESRP1 ectopic expression significantly downregulated CD44 overall expression		Human melanoma cell line MDA-MB-435 Prasad and Gopalan (2015)	Warzecha et al. (2009a)
	ESRP1 knockdown caused no effects on the expression level of CD44 transcripts		Human melanoma cell line Lu1205M	Zhang et al. (2016)
	Promotes exon v5 inclusion in the CD44 minigene splicing reporter system		Human embryonic kidney cell line HEK293FT	Harvey et al. (2018)
ESRP1 and ESRP2	Simultaneous depletion of ESRP1 and ESRP2 significant decreased the inclusion of CD44 variant exons (mainly exons v8–v10) and increased expression of CD44 isoform 4	• Increased expression of the mesenchymal isoforms of p120-catenin and FGFR2	Normal human prostatic epithelial cell line PNT2	Warzecha et al. (2009a)
		• Silencing epithelial-specific isoform of ENAH.		
	Simultaneous depletion of ESRP1 and ESRP2 led to switching from CD44 isoform 4 to variant isoforms		Human mammary epithelial cell line HMLE	Warzecha et al. (2009a)
ESRP2	ESRP1 knockdown caused no effects on the expression of CD44 isoforms	Enhanced cell motility	Human head and neck squamous cell carcinoma cell lines SAS and HSC4	Ishii et al. (2014)
hnRNPF	Promotes exon v8 inclusion in the CD44 minigene splicing reporter system		Human embryonic kidney cell line HEK293FT.	Hu et al. (2020)
hnRNPL	Promotes exon v10 skipping in *CD44* mRNA.		Human breast cancer cell line MDA-MB-231	Loh et al. (2015)
	hnRNPL knockdown increased exon v10 skipping in the CD44 minigene system		Human colorectal cancer cell line HCT-116	Loh et al. (2015)
hnRNPLL	hnRNPLL knockdown increased the expression of CD44 isoforms containing exons v3-v10	Increased invasion activity of human colon cancer cells	Human colon cancer cell line SW480, mouse colon cancer cell line CMT93	Sakuma et al. (2018)
	hnRNPLL knockdown increased the expression of CD44 isoforms containing exon v6	Significantly more metastatic nodules	Mouse colorectal cancer CMT93 cell line	Sakuma et al. (2018)
hnRNPM	Promotes variant exons skipping, switching form CD44 isoform 4 to isoform containing variant exons (including exon v6, v8, v8-v9, and v5-v6)	hnRNPM knockdown completely abolished TGFβ-induced CD44 isoform switching from variant isoforms to isoform 4 and inhibited TGFβ-induced EMT.	Human breast cancer cell lines LM2 (MDA-MB-231 derivatives cells), TGFβ-inducted mesenchymal MCF10A (Mes10A), and MCF-7; human mammary epithelial HMLE cells; murine breast cancer T4 cells	Xu et al. (2014), Sun et al. (2017), Zhang et al. (2018)
	Promotes variant exons v5 and v8 skipping in the CD44 minigene splicing reporter system	• The presence of AKAP8 dampened the effect of hnRNPM on promoting CD44 exon v8 skipping	Human embryonic kidney cell lines HEK293 and HEK293FT	Xu et al. (2014), Harvey et al. (2018), Hu et al. (2020)
		• AKAP8 silencing led to a more drastic effect of hnRNPM on exon skipping in both CD44v8 and CD44v5 minigenes		
	• Caused no effects on CD44 exon v8 skipping in the CD44 minigene splicing reporter system		Human colorectal cancer cell line HCT-116	Xu et al. (2014)
	• ESRP1-knockdown in HCT116 cells restores hnRNPM’s ability to promote exon skipping			
hnRNPR	Promotes exon v8 skipping in the CD44 minigene splicing reporter system		Human embryonic kidney cell line HEK293FT	Hu et al. (2020)
MBNL3	MBNL3 knockdown increased the expression of variant CD44 isoform 3	An activation of a pluripotency network	Human acute myeloid leukemia stem cells	Holm et al. (2015)
NONO	• Promotes the inclusion of exons v4-v5 in the CD44 minigene splicing reporter system		Human cervical carcinoma HeLa cell line	Zhao et al. (2022)
	• Overexpression on NONO caused no effects on CD44 transcripts levels in cells			
	Promotes the inclusion of exons v4-v5 in the CD44 minigene splicing reporter system		Human embryonic kidney HEK293T cells	Liu et al. (2011)
NSrp70	NSrp70 overexpression increased exon v5 inclusion in the CD44 minigene splicing reporter system	NSrp70 counteracts SRSF1- and SRSF2-induced CD44 exon v5 exclusion	Human embryonic kidney cell line HEK293T	Kim et al. (2011b), Kim et al. (2016)
PCBP1	Promotes skipping of variant exons v3, v5, v6, v8, and v10 exons (but not exon v9) in *CD44* mRNA	• PCBP1 overexpression decreased cell invasion	Human hepatoma cell line HepG2	Zhang et al. (2010)
		• PCBP1 knockdown increased cell invasion		
PTBP1	PTBP1 knockdown decreased the expression of CD44 isoforms containing exon v6	• M16 cells showed a significant decrease in cell migration	Human melanoma brain metastases’ cell lines BD and M16	Marzese et al. (2015)
		• BD cells showed a significant increase in cell migration		
	Promotes exon v8 inclusion in the CD44 minigene splicing reporter system		Human embryonic kidney cell line HEK293FT	Hu et al. (2020)
QKI	Negative regulator of CD44 isoform 3 formation (bioinformatic prediction)		Tumor samples of patients with colorectal cancer	Novosad (2023)
RBFOX2	RBFOX2 knockdown caused no effects on the inclusion of exons v8-v10 in *CD44* mRNA.		Mouse non-transformed mammary epithelial cell line NMuMG and epithelial murine breast cancer cell line PY2T	Braeutigam et al. (2013)
	Negative regulator of variant exon inclusion in *CD44* mRNA (bioinformatic prediction)		Colon adenocarcinoma samples of patients	Danan-Gotthold et al. (2015)
RBFOX2 and ESRP1	Upregulation of long transcript variant of RBFOX2 and downregulation of short variant of RBFOX2 and ESRP1 in response to ectopic expression of WNT5A downregulated inclusion of exons v4-v6 (but not v9) in *CD44* mRNA	• Reduced cell migration	Mouse breast cancer cell line 4T1	Jiang et al. (2013)
		• Less lung metastasis		
RBM3	Promotes switching from variant CD44 isoform 3 to standard CD44 isoform 4	RBM3 overexpression attenuated CSC features of prostate cancer cells and reduced tumor formation in nude mice	Human prostate adenocarcinoma cell line PC3	Zeng et al. (2013)
RBM10	Promotes exon v8 skipping in the CD44 minigene splicing reporter system		Human embryonic kidney cell line HEK293FT	Hu et al. (2020)
RBMX	Increased exon v8 skipping in the CD44 minigene splicing reporter system		Human embryonic kidney cell line HEK293FT	Hu et al. (2020)
Sam68	Sam68 knockdown decreased the expression level of CD44 variant isoforms		Human cervical carcinoma HeLa cell line	Cheng and Sharp (2023)
	Sam68 overexpression increased exon v5 inclusion in the CD44 minigene splicing reporter system after treatment with phorbol ester		Mouse EL4 T-lymphoma cells	Matter et al. (2002)
	Sam68 overexpression increased exon v5 inclusion in the CD44 minigene splicing reporter system	Simultaneous overexpression of SND1 led to a synergic effect with Sam68 on variant exon inclusion	Human embryonic kidney cell line HEK293T	Cappellari et al. (2013)
	Sam68 knockdown decreased the inclusion of variable exons v4, v5, v7, v8, v9, v10 in *CD44* mRNA (especially the exons v4, v5 and v7)	Reduced proliferation and migration of prostate cancer cells	Human prostate adenocarcinoma cell line PC3	Cappellari et al. (2013)
	Sam68 knockdown caused no effects on the expression level of CD44 transcripts		Human melanoma cell line Lu1205M	Zhang et al. (2016)
SFPQ	Promotes the skipping of exons v4-v5 in the CD44 minigene splicing reporter system		Human embryonic kidney HEK293T cells	Liu et al. (2011)
	• Promotes the inclusion of variant exons v4-v5		Human cervical carcinoma HeLa cell line	Zhao et al. (2022)
	• Overexpression on SFPQ caused no effects on CD44 transcripts levels in cells			
	SFPQ knockdown reduced the expression of CD44 isoforms containing exon v6	• Inhibition of cell stemness	Human lung cancer mesenchymal stem cells isolated from lung tissue biopsies	Yang et al. (2022)
		• Inhibition of cell proliferation *in vitro*		
		• Reduction of metastasis in mice		
SRm160	SRm160 knockdown decreased the expression of CD44 variant isoforms	Decrease in HeLa cell invasiveness	Human cervical carcinoma HeLa cell line	Cheng and Sharp (2023)
	SRm160 knockdown caused no effects on the expression level of CD44 transcripts		Human melanoma cell line Lu1205M	Zhang et al. (2016)
SRp20	SRp20 knockdown caused no effects on the expression level of CD44 transcripts		Human melanoma cell line Lu1205M	Zhang et al. (2016)
SRSF1	Positive regulator of CD44 isoform 3 (but not isoforms containing exon v6 or exons v6-v10) expression, switching from CD44 isoform 3 to isoform 4		Human gastric carcinoma cell line MGC-803	Peng et al. (2019)
	• Promotes exon v6 skipping in the CD44 minigene splicing reporter system		Human breast cancer MCF7	Loh et al. (2016)
	• SRSF1 knockdown decreased the expression of CD44v6-v10 and CD44v6,v8-v10 isoforms in cells			
	Promotes exon v5 skipping in the CD44 minigene splicing reporter system		Human embryonic kidney cell line HEK293T	Kim et al. (2016)
SRSF2	• Promotes exon v6 skipping in the CD44 minigene splicing reporter system		Human breast cancer MCF7	Loh et al. (2014)
	• SRSF2 knockdown decreased the expression of CD44v6 isoform but increased the expression of CD44v6-v10 and CD44v6,v8-v10 isoforms in cells			
	Promotes exon v5 skipping in the CD44 minigene splicing reporter system		Human embryonic kidney cell line HEK293T	Kim et al. (2016)
SRSF3	• Caused no effects on exon v6 splicing in the CD44 minigene splicing reporter system		Human breast cancer MCF7	Loh et al. (2014)
	• SRSF3 knockdown decreased the expression of CD44v6-v10 and CD44v6,v8-v10 isoforms in cells			
	Positive regulator of CD44 variant isoforms expression	The reduction of CD44 variant isoform expression due to SRSF3 silencing could be partially rescued through the elevation of TDP43	Human triple-negative breast cancer cell lines HCC1806 and MDA-MB-231	Guo et al. (2022a)
SRSF4	• Caused no effects on exon v6 splicing in the CD44 minigene splicing reporter system		Human breast cancer MCF7	Loh et al. (2014)
	• SRSF4 knockdown caused no effect on the expression level of CD44 transcripts in cells			
SRSF6	SRSF6 overexpression increased exon v6 skipping in the CD44 minigene splicing reporter system		Human breast cancer MCF7	Loh et al. (2016)
SRSF9	• SRSF9 overexpression increased exon v6 skipping in the CD44 minigene splicing reporter system		Human breast cancer MCF7	Loh et al. (2016)
	• SRSF9 knockdown caused no effects on expression levels of endogenous CD44 transcripts			
	• SRSF9 overexpression increased exon v10 skipping in the CD44 minigene splicing reporter system		Human embryonic kidney cell line HEK293T and colorectal cancer cell line HCT116	Oh et al. (2020)
	• SRSF9 knockdown caused no effects on exon v10 splicing in endogenous *CD44* mRNA			
TDP43	Promotes the inclusion of variant exons in *CD44* mRNA, especially exons v8, v9, and v10	TDP43 knockdown reduced stemness features of breast cancer stem cells	Human triple-negative breast cancer cell lines HCC1806 and MDA-MB-231	Guo et al. (2022a)
Tra2β	• Tra2β overexpression increased exon v10 inclusion in the CD44 minigene splicing reporter system		Human embryonic kidney cell line HEK293T and colorectal cancer cell line HCT116	Oh et al. (2020)
	• Tra2β knockdown caused no effects on exon v10 splicing in endogenous *CD44* mRNA.			
	Tra2β knockdown caused no effects on the expression level of CD44 transcripts		Human melanoma cell line Lu1205M	Zhang et al. (2016)
U2AF2	Promotes switching from standard CD44 isoform 4 to variant CD44 isoform 3	U2AF2 knockdown diminished the adhesion probability of Lu1205M cells and reduced the number of metastatic lesions	Human melanoma cell lines Lu1205M and SK-Mel-25	Zhang et al. (2016)
YB-1	Increased the inclusion of variant exons v4 and v5 in the CD44 minigene splicing reporter system		Human cervical carcinoma HeLa cell line	Stickeler et al. (2001)
	YB-1 knockdown caused no effects on the expression level of CD44 transcripts		Human melanoma cell line Lu1205M	Zhang et al. (2016)
ZMAT3	ZMAT3 knockdown increased the expression of CD44 variant isoforms 1 and 2 with a concomitant reduction of standard CD44 isoform 4	Increase in clonogenicity of tumor cells	Human colorectal cancer cell line HCT116	Muys et al. (2021)

## 4 RNA-binding proteins regulating CD44 alternative splicing

**FIGURE 3:**
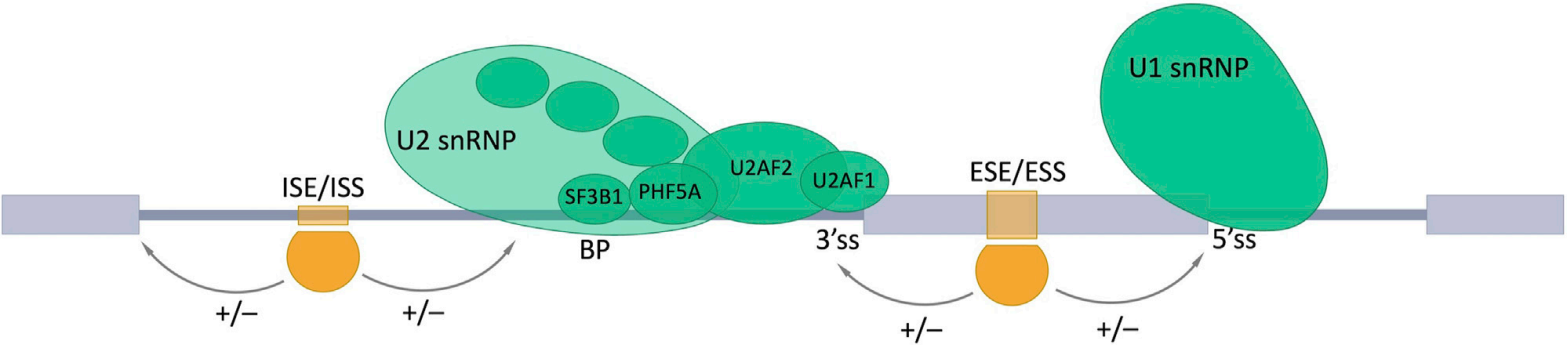
Involvement of RNA-binding proteins in the alternative splicing (AS). AS is regulated by combined action of trans- and cis-acting elements. Trans-acting elements are represented by different RBPs (shown as orange shapes). Cis-acting elements are specific nucleotide motifs in pre-mRNA: intronic and exonic splicing enhancers (ISE and ESE), which promote the inclusion (+) of the AS exon by providing the binding sites for activators (shown in orange); intronic and exonic splicing silencers (ISS and ESS) are bound by repressors (shown in orange) and promote exon skipping (−). Exons are represented as gray boxes, introns as gray lines. BP, a branch point; 3’ss, 3′ splice site; 5’ss, 5′ splice site.

RNA binding proteins (RBPs) recognize and bind target sequences in pre-mRNAs, which allows for the inclusion or skipping of alternative exons. Such target sequences could be intronic or exonic splicing enhancers (ISEs or ESEs) or intronic or exonic splicing silencers (ISSs or ESSs), which nucleate the assembly of complexes of regulatory factors that promote or inhibit splice site recognition by the core splicing machinery (Ule and Blencowe, 2019; Rogalska et al., 2022) (Figure 3).

### 4.1 ESRP1 and ESRP2

**FIGURE 4:**
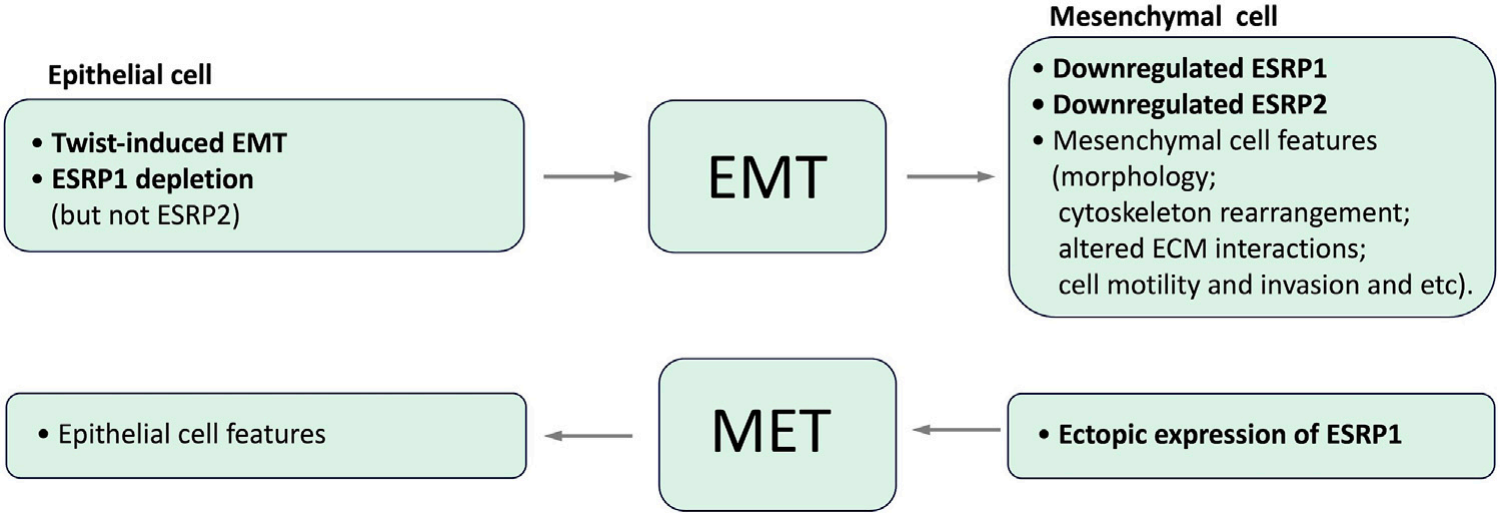
Interdependence between expression of ESRPs and EMT.

Epithelial splicing regulatory proteins (ESRPs), including ESRP1 and ESRP2 (also known as RBM35A and RBM35B, respectively), are specifically expressed in epithelial cells and identified as core modulators of EMT-related splicing events (Mashita et al., 2014; Katsuno and Derynck, 2021; Liu et al., 2022). In particular ESRP1 and ESRP2 regulate the alternative splicing of a number of proteins important for maintaining an epithelial phenotype. It has been shown that *ESRP1* transcription is regulated by Snail and ZEB1, EMT-related transcription factors, through its direct binding to *ESRP1* promoter (Reinke et al., 2012; Preca et al., 2015; Chen et al., 2017). Induction of EMT results in the downregulation of ESRP1 and ESRP2, whereas the depletion of ESRP1, but not ESRP2 (Warzecha et al., 2009b), is sufficient to induce mesenchymal splicing patterns (Warzecha et al., 2009b; Jeong et al., 2017). Ectopic expression of ESRP1/mouse Esrp1 in human mesenchymal cells induces epithelial-specific changes in the splicing of ESRP1 target transcripts (Warzecha et al., 2009a; Warzecha et al., 2009b). Interdependence between expression of ESRPs and EMT is summarized in Figure 4.

Numerous studies implicate ESRP1 as a regulator of CD44 isoform switching (see below, Table 2). It has been reported that ESRP1 recognizes GGU/UGG–rich sequences (Dittmar et al., 2023). In particular it has been shown that ESRP1 promotes the inclusion of CD44 variant exon v8 by directly binding to the GGU/UGG–rich motifs located in the intron downstream of this exon (Reinke et al., 2012). Another study indicated the dependence of ESRP1 and ESRP2 upon the presence of ISE/ISS-3 and/or the UGCAUG motif to promote splicing (Warzecha et al., 2009a).

Simultaneous depletion of ESRP1 and ESRP2 in the normal human prostate epithelium cell line PNT2 causes a significant decrease in the inclusion of CD44 variant exons (mainly exons v8–v10 corresponding to CD44 isoform 3) and an increase in the standard CD44 isoform 4 in which all the variable exons are skipped (Warzecha et al., 2009a; Warzecha et al., 2009b). This isoform skipping was also accompanied by increased expression of the mesenchymal isoforms of p120-catenin and FGFR2 and silencing epithelial-specific isoform of ENAH.

The loss of ESRPs expression in the human mammary epithelial cell line HMLE due to the induction of an EMT by the transcription factor Twist resulted in the elevated level of CD44 standard isoform 4 expression and the decreased CD44 variant isoforms expression, indicating codependence of these events (Warzecha et al., 2009a). ShRNA-mediated depletion of ESRP1 in HMLE cells resulted in a shift of expression from CD44 variant isoforms to isoform 4, with only a slight effect on the overall CD44 expression level and accelerated EMT (Brown et al., 2011), while ESRP1 overexpression regulated CD44 alternative splicing in the opposite direction and inhibited Snail-induced EMT in these cells (Reinke et al., 2012). Notably, this isoform switch from CD44 variant isoforms to isoform 4 was essential for forming breast tumors in mice (Brown et al., 2011).

To test whether ectopic expression of the ESRP1 in mesenchymal cells would restore an epithelial splicing pattern, the mesenchymal human breast cancer cell line MDA-MB-231 was transduced with virus encoding FLAG-tagged ESRP1 (Warzecha et al., 2009a). The expression of ESRP1 caused a switch from primarily CD44 isoform 4 expression to variant isoforms (including CD44 isoform 3 and CD44v6-v10). Interestingly, the repeat of the experiment with mesenchymal melanoma cell line MDA-MB-435 (Prasad and Gopalan, 2015) with predominant expression of the standard CD44 isoform 4 did not lead to the same results, overall expression of CD44 was significantly downregulated (Warzecha et al., 2009a). The same authors ectopically expressed mouse Esrp1 in human MDA-MB-231 cells and also showed the increased inclusion of human CD44 variant exons and the decreased expression of human CD44 isoform 4 (Warzecha et al., 2009b). ESRP1 downregulation in 4T1 mouse breast cancer cells also resulted in an isoform switch from CD44 containing variant exons to CD44 isoform 4 (Yae et al., 2012). However, this downregulation led to suppression of lung colonization, presumably due to reduced cell surface expression of the cystine transporter xCT, the stability of which is controlled by CD44 variant isoforms. A more recent study demonstrated that ectopic expression of ESRP1 inhibits the production of standard CD44 isoform 4 and thus inhibits CSC properties of TGFβ-treated HMLE cells, where endogenous ESRP1 expression was low (Zhang et al., 2019). In contrast, ESRP1-depletion possessed enhanced mammosphere-forming ability of TGFβ-treated HMLE cells, and silencing CD44 in these ESRP1-depleted cells abrogated mammosphere formation. In addition, Zhang et al. expressed ESRP1 in a mesenchymal triple-negative breast cancer cell line SUM159 and revealed reduced potential for mammosphere formation, which was rescued by coexpression of CD44 isoform 4 but not CD44v3-v10 (Zhang et al., 2019). A transient knockdown of ESRP1 in human breast cancer MCF7 and human pancreatic adenocarcinoma BxPC-3 cells resulted in a shift of expression from CD44v (containing exon v6) to CD44 isoform 4 without affecting total CD44 level (Preca et al., 2015). Vice versa, overexpression of ESRP1 in undifferentiated, mesenchymal breast cancer MDA-MB231 cells and pancreatic ductal adenocarcinoma Panc-1 cells resulted in a reverse isoform switch accompanied by decreased ZEB1 levels (Preca et al., 2015).

Silencing of ESRP1 significantly decreased the expression of CD44 isoforms containing exon v6 in human MB and LH cells derived from melanoma lymph node metastases (Marzese et al., 2015). These siESRP1-transfected melanoma cells also demonstrated lower migratory potential under hepatocyte growth factor (HGF) treatment (HGF, a factor released during inflammation or tissue disruption, should increase the migration of CD44v6-positive melanoma cells). Further in this study, it has been shown that ESRP1 is epigenetically silenced in human melanoma brain metastasis, and high expression of CD44 containing exon v6 in early stages is a significant predictor of melanoma brain metastasis development (Marzese et al., 2015).

Switching from CD44 variant isoforms containing exon v7 to isoform 4 without change in a total amount of CD44 was observed in human epithelial ovarian cancer cell line HO8910 with a stable suppression of ESRP1 expression (Chen et al., 2017). This switch in expression was accompanied by increasing migratory and invasive capabilities of the ESPRP1 suppressed cells. Moreover, the siRNA-mediated downregulation of CD44 expression, in turn, suppressed migration and invasion of the ESRP1-depleted HO8910 cells, indicating that ESRP1 suppresses HO8910 cell motility mainly by repressing CD44 isoform switching (Chen et al., 2017). An enforced ESRP1 expression in the ovarian cancer cell line SKOV3, significantly reduced the level of the mesenchymal cell-specific CD44 isoform 4 and increased levels of CD44 variant isoforms as well as caused overall switching from mesenchymal to epithelial phenotype of cells (Jeong et al., 2017).

Based on the qPCR measurement in 14 colorectal cancer (CRC) cell lines, it has been demonstrated a higher *ESRP1* expression was noted in epithelial phenotype cells than those of mesenchymal phenotype (Mashita et al., 2014). The higher *ESRP1* expression strongly correlated with higher expression of CD44 variant exon v9 and lower expression of CD44 isoform 4, respectively. An inverse correlation between the expression of ESPRs and CD44 alternative splicing was observed for CRC cell line LS1034 on mRNA level (Dinger et al., 2020). In particular LS1034 xenografted cancer cells demonstrated an elevated expression of *CD44* mRNA isoform 3 compared to cultured cells, whereas *ESPR1* expression was reduced and *ESRP2* expression was essentially enhanced in the LS1034 xenografts. However, ESRP1 silencing in HCT-116 cell line has shown suppressed *CD44* mRNA variant isoforms and enhanced standard isoform 4 expressions, which were accompanied by inducing caspase-independent cell death (Vadlamudi and Kang, 2022).

Higher *ESRP1* expression was associated with CD44 variant isoforms, including CD44 isoform 1 (CD44v2-v10), isoform 3 (CD44v8-v10), and CD44v6-v10, in human head and neck squamous cell carcinoma cell lines (Ishii et al., 2014). Si-RNA-mediated silencing of ESRP1 in SAS and HSC4 cells resulted in switching from the CD44 variant isoforms to the CD44 standard isoform 4 (Ishii et al., 2014). In contrast, silencing of ESRP2 did not affect CD44 isoform switching. Although knockdown of both ESRPs enhanced cell motility without effect on cell proliferation. Further, it has been shown that ESRPs suppress cell motility in HNSCC through distinct mechanisms: ESRP1 regulates the dynamics of the actin cytoskeleton through repressing expression of the Rac1b isoform, whereas ESRP2 is involved in the regulation of cell-cell adhesion by suppressing EMТ-associated transcription factors (Ishii et al., 2014).

Several splice variants of ESRP1 were tested for their ability to regulate CD44 alternative splicing (Kim et al., 2018). Overexpression of ESRP1 v1, v4, or v5 in fully differentiated human foreskin fibroblasts resulted in converting CD44v9-v10 to CD44v7-v10. ESRP1 knockdown in undifferentiated H9 human embryonic stem cell induced downregulation of CD44v7-v10 expression as well as the loss of pluripotency of the cells. Thus, regulating the ESRP1-CD44v7-v10 axis is crucial for human pluripotency maintenance and reprogramming of human somatic cells to pluripotent stem cells (iPSCs) (Kim et al., 2018).

Overall, the presented information indicates that ESRP1 is mainly a positive regulator of including variant exons (many of them) in CD44 transcript, whereas ESRP2 is apparently not so much involved in CD44 alternative splicing.

### 4.2 RBFOX2 and QKI

RBFOX2 is a member of the RNA-binding Fox (RBFOX) protein family (RBFOX1, RBFOX2 and RBFOX3) regulating alternative splicing (Kuroyanagi, 2009). RBFOX1 is expressed in heart, skeletal muscle and neuronal tissues, whereas RBFOX2 is ubiquitously expressed in many tissues from the embryonic-stem-cell stage through adulthood (Jin et al., 2003; Yeo et al., 2009; Kim K. K. et al., 2011; Underwood et al., 2023). RBFOX3 is expressed exclusively in the brain (Kim et al., 2009). All three proteins contain a single conserved RNA-recognition motif (RRM) and recognize the consensus sequence (U)GCAUG in the introns flanking target exons. RBFOX2 prevents the binding of U2AF2 to the 3′-splice site (Ivanova et al., 2023). A general rule for RBFOX2-regulated exon inclusion or skipping in a position-dependent manner has been revealed. In particular, RBFOX2 promotes exon skipping when it binds upstream of the alternative exon but inclusion occurs when it binds downstream of this exon (Yeo et al., 2009). Of note, *RBFOX2* mRNA undergoes extensive alternative splicing itself, thus generating many isoforms with a common RRM. The RBFOX2 splice variants show differences in intracellular localization and splicing activity (Nakahata and Kawamoto, 2005). Within the nucleus RBFOX2 can operate their targets using three binding modes: single, multiple or secondary (Damianov et al., 2016; Zhou et al., 2021). In the single binding mode, RBFOX2 is recruited to its target splice sites through a single canonical binding motif, while in the multiple binding mode, RBFOX2 binding sites include the adjacent binding of at least one other RBP partner. In the secondary binding mode, RBFOX2 is recruited to splice sites lacking its canonical binding motif by binding one of its protein partners and likely without direct binding to mRNA. Interestingly, many targets of RBFOX2 are themselves splicing regulators (Yeo et al., 2009). In addition, RBFOX2 is implicated in the biogenesis of some miRNAs (e.g. miR-20b and miR-107) and, thus, in the expression of their downstream targets (Chen et al., 2016). All of these different modes of operation may explicate an ambiguous role of RBFOX2 in cancer progression. Thus, several studies have reported that RBFOX2 is important to specify a mesenchymal splicing signature in breast (Braeutigam et al., 2013), colon and ovarian tissues (Venables et al., 2013). RBFOX2 promotes oncogenic splice-switching and the resulting mesenchymal signature and drives an invasive phenotype in breast cancer (Braeutigam et al., 2013; Ahuja et al., 2020). However, other studies have reported the anti-metastatic role of RBFOX2 in pancreatic cancer (Jbara et al., 2023) and its decreased expression in breast, colon, and prostate adenocarcinomas (Danan-Gotthold et al., 2015), as well as ovarian cancer (Venables et al., 2009). In general, RBFOX2 is considered a mesenchymal marker (Pradella et al., 2017; Lambert and Weinberg, 2021), whereas it also promotes epithelial-specific splicing in some cases (Braeutigam et al., 2013; Baraniak et al., 2023). A possible clue of these contradictions may be that the epithelial state of cells is determined by the ratio of the expression levels of RBFOX2 and ESRP1 (Barriere et al., 2014; Meng et al., 2019).

Based on the analysis of patient TCGA RNA-Seq data of colon adenocarcinoma and corresponding normal colon, RBFOX2 has been predicted to act as a negative regulator of variant exon inclusion in *CD44* mRNA (Danan-Gotthold et al., 2015). Interestingly, it was not the case in seven other analyzed cancer types (breast invasive carcinoma, kidney clear cell carcinoma, liver hepatocellular carcinoma, lung adenocarcinoma, prostate adenocarcinoma, head and neck squamous cell carcinoma, and thyroid carcinoma). Our bioinformatic analysis of mRNA-Seq data of 56 colorectal cancer cell lines downloaded from the CCLE database revealed the association of higher expression of RBFOX2 with a higher level of CD44 isoform 4 and a lower level of CD44 isoform 3 (Novosad and Maltseva, 2023).

Si-RNA mediated knockdown of RBFOX2 did not alter the level of inclusion of exons v8-v10 into *CD44* mRNA in both non-transformed mammary epithelial cell line NMuMG and epithelial murine breast cancer cell line PY2T under normal conditions and TGF-β-treatment (Braeutigam et al., 2013). Upregulation of the long transcript variant of RBFOX2 and downregulation of short variant RBFOX2 and ESPR1 in mouse breast cancer 4T1 cell line in response to ectopic expression of WNT5A (a non-canonical Wnt signaling) was accompanied by downregulated inclusion of exons v4-v6 (but not v9) in *CD44* mRNA (Jiang et al., 2013). These events also were associated with reduced cell migration and fewer spontaneous lung metastasis.

It has been observed that RBFOX2 co-operates with Quaking (QKI) in the splicing regulation of common pre-mRNA targets (Brosseau et al., 2014; Danan-Gotthold et al., 2015; Yang et al., 2023). QKI is an RBP belonging to the signal transduction and activation of RNA (STAR) protein family, which binds specifically to RNA containing ACUAA motifs (Hall et al., 2013). QKI regulates several posttranscriptional processes, including AS, mRNA localization, mRNA stability, and protein translation (Saccomanno et al., 1999; Li et al., 2000; Larocque et al., 2002; Zhao et al., 2010; Hall et al., 2013). Among the three major isoforms of QKI (QKI-5, QKI-6, and QKI-7), only QKI-5 is predominantly localized in the nucleus and is involved in the regulation of AS (Ebersole et al., 1996; Bockbrader and Feng, 2008). QKI binding in the downstream intron promotes exon inclusion while binding in the upstream intron promotes exon skipping (Hall et al., 2013). Of note, QKI strongly induces the mesenchymal and stem-like phenotypes (Li et al., 2018; Mukohyama et al., 2019) and promotes mesenchymal splicing patterns (Lambert and Weinberg, 2021; Yang et al., 2023).

To our knowledge, the involvement of QKI in CD44 splicing regulation has yet to be experimentally confirmed. However, such a possibility was predicted for colorectal cancer based on bioinformatic analysis of mRNA-Seq data of patient tumor samples (Novosad, 2023). In particular QKI has been shown as a potential negative regulator of CD44 isoform 3 formation, what is consistent with the EMT-promoting role of QKI.

### 4.3 NONO and its protein partners

Non-POU domain-containing octamer-binding protein (NONO, also known as p54nrb) belongs to the Drosophila behavior/human splicing (DBHS) family (Knott et al., 2016). In humans, the DBHS protein family also includes two other members: splicing factor proline/glutamine-rich (SFPQ, also known as PSF) and paraspeckle protein component 1 (PSPC1, also known as PSP1). All three DBHS proteins contain two highly conserved RNA-recognition motifs and a nuclear localization signal and are regarded mainly as nuclear factors. However, they may additionally function intra-cytoplasmically and on the cell surface (Knott et al., 2016). Structural and biological data suggest that DBHS proteins rarely function alone. They are found in the nucleoplasm within the subnuclear domain termed paraspeckles (Knott et al., 2016), which are known to regulate RNA metabolism, including splicing, stabilization and export, as well as DNA repair (Wang and Chen, 2020). NONO and SFPQ were found to be associated with both the hypophosphorylated and hyperphosphorylated forms of RNA polymerase II in HeLa cell extracts, indicating that these two proteins could provide a direct physical link between RNA polymerase and other pre-mRNA processing components (Emili et al., 2002).

NONO is a multipurpose protein engaging in almost every step of gene regulation, including transcriptional activation and inhibition, RNA processing, and DNA repair (Knott et al., 2016). Dysregulation of NONO has been found in many types of cancer entities (Feng et al., 2020). In some of them, such as bladder cancer, lung cancer, prostate cancer, and oesophageal squamous cell carcinoma, glioblastoma multiforme NONO exhibits tumor promoting role, as it induces cell proliferation and inhibits apoptosis. In contrast, in estrogen receptor-negative breast cancer, it demonstrates tumor suppressive functions (Feng et al., 2020; Wang et al., 2022). Recently, it has been shown that NONO induces expression of ZEB1 and CD44 in LN229 glioblastoma cells and patient-derived P3glioblastoma stem-like cells and promoted cells migration and invasion indicating an association between NONO and EMT (Wang et al., 2022). Si-RNA-mediated loss of NONO in U251 and P3 glioblastoma cells reduced levels of proteins involved in the EMT but increased those involved in apoptosis (Wang et al., 2022). Silencing of NONO inhibits EMT and stemness of breast cancer cells, as well as its growth, survival, migration and invasion (Lone et al., 2023).

Using the CD44 minigene reporter system [the variant exons v4 and v5 of the human CD44 gene, along with their surrounding intron sequences, inserted into an intron of the β-globin gene driven by the HSV promoter (Auboeuf et al., 2002)] it has been demonstrated that NONO and SFPQ regulate alternative splicing of CD44 variant exons in HEK293T cells transfected with either NONO vector or SFPQ vector (Liu et al., 2011). NONO decreased the ratio of skipping to inclusion of CD44 exons v4-v5, whereas SFPQ increased this skipping-inclusion ratio (Liu et al., 2011). Interestingly, dephosphorylation of NONO and SFPQ by protein phosphatase 1 (PP1) reduced their alternative splicing activity on CD44 minigene.

Knockdown of SFPQ in lung cancer mesenchymal stem cells resulted in the reduced expression of CD44 isoforms containing exon v6 with concomitant inhibition of cell stemness, proliferation *in vitro*, and metastasis *in vivo* (Yang et al., 2022).

Applying the CD44 minigene reporter system by Zhao et al. showed that overexpression of both NONO and SFPQ in HeLa cells significantly increases variant exon inclusion and decreases a level of CD44 isoform 4 (Zhao et al., 2022). Of note, a simple overexpression of NONO or SFPQ in HeLa cells did not significantly affect the transcription level of neither CD44 isoform 4 nor CD44 variant isoforms. In the same work, the authors revealed the interaction of NONO and SFPQ with an angiogenic factor AGGF1 in paraspeckles, which forms an outside rim around the NONO/SFPQ/PSP1 core (Zhao et al., 2022). Interestingly, the overexpression of AGGF1 in HeLa cells in turn resulted in enhanced inclusion of exons v4 and v5 (but not v8-v10 or v10) in CD44 mRNA and decreased level of CD44 isoform 4. The enhanced inclusion of exons v4 and v5 was also detected in HeLa cells co-transfected with CD44 minigene reporter and AGGF1 vector. Also, Zhao et al. detected the decreased ratio of skipping to inclusion of exons v4-v5 in the CD44 minigene in response to overexpression of DHX15 (DEAH-Box Helicase 15), interacting with AGGF1 in HeLa cells (Zhao et al., 2022). Co-overexpression of AGGF1 with NONO, SFPQ, or DHX15 also enhanced the inclusion of exons v4 and v5 in the CD44 minigene.

Thus, the available evidence suggests that NONO, AGGF1, and DHX15 function primarily as an enhancer of the formation of variant CD44 isoforms. At the same time, SFPQ can contribute to both the skipping and inclusion of variant exons in the *CD44* transcript.

There is evidence for the interactions of NONO and SFPQ with the ubiquitously expressed heterogeneous nuclear ribonucleoprotein M (hnRNPM) and for the presence of the last one within a subpopulation of paraspeckles (Marko et al., 2010). hnRNPM is a component of the spliceosome machinery and can influence both constitutive and alternative splicing. It typically binds to ESS motives, thus antagonizing the recognition of splice sites and suppressing pre-mRNA splicing (Wahl et al., 2009). hnRNPM is associated with aggressive breast cancer and correlates with increased CD44s in patient specimens (Xu et al., 2014; Sun et al., 2017). Moreover, it has been demonstrated that hnRNPM precisely controls CD44 splice isoform switching during EMT and acts in a mesenchymal-specific manner in breast cancer cells (Xu et al., 2014; Harvey et al., 2018). Silencing hnRNPM completely abolished TGFβ-induced CD44 isoform switching from CD44 variant isoforms to isoform 4 in HMLE cells (Xu et al., 2014). The hnRNPM depletion was also accompanied by a general inhibition of TGFβ-induced EMT in HMLE cells which resulted in the reduction of spontaneous lung metastasis numbers in mice with into the mammary fat pad implanted murine T4 breast cancer cells. Reduced dissemination potential of murine T4 breast cancer cells and human LM2 breast cancer cells (MDA-MB-231-derived lung metastatic cells) with hnRNPM knockdown was also shown after intravenous injection into murine tail vein (Xu et al., 2014). Interestingly, the enforced expression of CD44 isoform 4 overrode the loss of hnRNPM and permits EMT and metastasis formation to occur. In a combination of experiments with several cell lines, Xu et al. also demonstrated that hnRNPM is necessary and sufficient to stimulate CD44 variant exon skipping via its interaction with GU-rich motifs located in introns downstream from variable exons (Xu et al., 2014). In addition, a cell-type restricted activity of hnRNPM has been revealed as it does not promote CD44 exon skipping in HCT116 human colon cancer cells. A possible reason for this observation is that a competition of hnRNPM with ESRP1 for the binding to CD44 pre-mRNA exists (Xu et al., 2014). In their subsequent study, the authors showed that coregulation of alternative splicing by hnRNPM and ESRP1 is widespread and primarily antagonistic in breast cancer cells, although a subset of events is regulated concordantly (Harvey et al., 2018). An overexpression of hnRNPM in MCF-7 human breast cancer cells resulted in a decreased expression in CD44 isoforms containing exon v6 and an increased expression in CD44 isoform 4 with a slight change in the total level of CD44 transcripts (Sun et al., 2017). These changes in expression levels resulted in an increased invasion capacity of MCF-7 cells.

In triple-negative breast cancer cells, hnRNPM has been shown as a binding partner of a mutated form of chromatin regulatory protein MORC2 (microorchidia family CW-type zinc finger 2) (Zhang et al., 2018). The mutation of MORC2 protein consists in substitution of methionine to isoleucine at residue 276 (M276I), a cancer-associated mutation, which enhances the interaction of MORC2 with hnRNPM. This interaction promotes the hnRNPM-mediated splicing switch from the epithelial CD44 variant isoform containing exons v5/v6 to the mesenchymal CD44 isoform 4, ultimately driving EMT. ShRNA-mediated knockdown of hnRNPM reduced the binding of mutant MORC2 to CD44 pre-mRNA. It also reversed the mutant MORC2-induced CD44 splicing switch and EMT, consequently impairing the migration, invasion, and lung metastasis potential of mutant MORC2-expressing cells in mice.

Based on experiments with HMLE and HEK293FT cells, it has been demonstrated that hnRNPM’s splicing activity on CD44 variant exon skipping could be inhibited by the interaction of hnRNPM with AKAP8 (the A-kinase anchoring protein 8), a recently identified RNA-binding protein (Hu et al., 2020). Several observations let the authors speculate that AKAP8 binding to hnRNPM blocks hnRNPM from binding to its RNA targets. Firstly, the AKAP8-hnRNPM interaction became stronger upon RNase treatment. Secondly, depletion of AKAP8 promoted hnRNPM’s ability to bind its consensus RNA sequences and to stimulate exon skipping. Significantly, AKAP8 can bind its own RNA consensus sequences and prevent CD44 variant exon skipping, as well as the other EMT-associated alternative splicing. AKAP8 itself inhibits EMT and breast cancer metastasis to the lung. In the same study, 28 other hnRNPM-interacting splicing factors have been found (Hu et al., 2020). Among them, PTBP1 and hnRNPF promoted exon v8 inclusion in CD44 exon v8 splicing minigene reporter assay, whereas RBM10, RBMX, and hnRNPR promoted exon skipping.

Summarizing the current studies, we can conclude that hnRNPM promotes the exclusion of variant exons from CD44 pre-mRNA in breast cancer. However, the role of hnRNPM in regulating alternative splicing is more complex and may vary in different cell types.

### 4.4 SR proteins

The serine/arginine (SR)-rich protein family of RNA-binding proteins includes 12 members (SRSF1-12) in humans (Busch and Hertel, 2012; Wagner and Frye, 2021). The alternative nomenclature for SR proteins is presented in (Manley and Krainer, 2010). SR proteins play important roles in both alternative and constitutive splicing. As the regulator of constitutive splicing, they promote the binding of U1 snRNP to a 5′ splice site and the binding of U2 snRNP to a branch point in spliceosome assembly. In general, SR proteins are shown to antagonize hnRNP functions in alternative splicing. Of note, not all SR proteins promote splicing. Thus, depending on their phosphorylation state, SRSF10 and SRSF12 also act as global splicing repressors (Wagner and Frye, 2021). SRSF1 has been described as a mesenchymal splicing factor (Lambert and Weinberg, 2021).

Two screening studies of SR proteins for CD44 splicing were performed by Loh et al. (2014); Loh et al. (2016). Overexpression of SR proteins in MCF7 cells stably expressing the pFlare-V6 plasmid (a kind of CD44 minigene reporter system containing CD44 variant exon v6) showed that SRSF3 and SRSF4 do not affect exon v6 splicing of *CD44* pre-mRNA, whereas SRSF1, SRSF6, SRSF9, and SRSF2 induced the exon v6 skipping. However, lentivirus-mediated shRNA treatment of MCF7 cells revealed that reduced expression of SRSF3 and SRSF1 caused a decrease of CD44v6-v10 and CD44v6,v8-v10 isoforms. Reduced expression of SRSF4 and SRSF9 did not induce a significant change in CD44 isoforms. Depletion of SRSF2 (also known as SC35) led to decreased expression of CD44v6 isoform but increased expression of both CD44v6-v10 and CD44v6,v8-v10 isoforms (Loh et al., 2016). These results indicate that CD44 minigene reporter systems could be used for the identification of RBPs’ responsive elements in exons or their flanking introns, but the endogenous regulation mechanisms of CD44 alternative splicing are more complicated in cells, and other events may play a role, e.g., the presence of other exons in *CD44* pre-mRNA. This conclusion is confirmed by the results obtained for HEK293 and HCT-116 cells (Oh et al., 2020). Thus, using a minigene-based approach, Oh et al. demonstrated the opposite roles of SRSF9 and Tra2β on CD44 variant exon v10 splicing. While SRSF9 inhibited exon v10 inclusion, Tra2β promoted exon v10 inclusion. They also showed that both proteins functionally bind to exon v10, in which SRSF9 recognizes the AAGAC sequence and Tra2β recognizes the GAAGAAG sequence. However, the knockdown of neither SRSF9 nor Tra2β did not affect endogenous CD44 exon v10 splicing in HEK293T and HCT116 cells.

In the triple-negative breast cancer cell lines HCC1806 and MDA-MB-231, SRSF3 has been identified as a positive regulator of variant exon inclusion in *CD44* pre-mRNA, especially exons v8, v9, and v10 (Guo L. et al., 2022). The loss of SRSF3 reduced the abundance of CD44 variant isoforms expression but increased the expression of CD44 standard isoform 4. Accordingly, exogenous expression of SRSF3 induced a significant increase in CD44 variant exon inclusion in the MDA-MB-231 and HCC1806 cells, while the total abundance of CD44 did not change. Interestingly, the reduction of CD44 variant isoform expression due to SRSF3 silencing could be partially rescued through the elevation of another splicing regulator TDP43 (TAR DNA-binding protein-43). Based on overexpression and knockdown experiments, it has been shown that TDP43 promotes variant exons inclusion in *CD44* mRNA, especially exons v8, v9, and v10, in triple-negative breast cancer cell lines MDA-MB-231 and HCC 1806 (Guo L. et al., 2022). The AS regulation occurs through the direct interaction of TDP43 with *CD44* pre-mRNA. The knockdown of TDP43 reduced stemness features of breast cancer stem cells. SRSF3, in turn, stabilized the *TDP43* mRNA by inhibiting non-sense-mediated decay and thereafter provides enough TDP43 proteins for the cooperative network to regulate the splicing of its target genes (Guo L. et al., 2022).

In MGC-803gastric cancer cells, the splicing of CD44 was controlled by SRSF1 (Peng et al., 2019). The depletion of SRSF1 led to a significant decrease in CD44 isoform 3 level (but not in isoforms containing exon v6 or exons v6-v10) and an increase in CD44 isoform 4 level. An overexpression of SRSF1, in turn, induced switching from CD44 isoform 4 to isoform 3.

The splicing activity of SRSF1 and SRSF2 could be counteracted by another SR protein family member namely by NSrp70 (Kim Y. D. et al., 2011; Kim et al., 2016). Based on the CD44 exon v5 minigene assay, it has been shown that NSrp70 and SRSF1/2 have opposite functions in HEK293T cells. The interaction of NSrp70 with SRSF1 and SRSF2 prevented the SRSF1- and SRSF2-induced CD44 exon v5 exclusion.

### 4.5 Other RNA-binding proteins

Several other RBPs have also been implicated in the splicing regulation of CD44. Based on siRNA-mediated knockdown, it has been shown that PTBP1, RBP recognizing CUCUCU-rich sequences (Oberstrass et al., 2005), induced a significant decrease in expression of CD44 containing exon v6 at mRNA and protein level in two melanoma brain metastases’ cell lines BD and M16 (Marzese et al., 2015). Interestingly, the reduction of PTBP1 affected the migration of BD and M16 cells treated with HGF in opposite directions: M16 showed a significant decrease, while BD showed a significant increase in cell migration. Also, PTBP1 promoted exon v8 inclusion in CD44 exon v8 minigene system in HEK293FT cells (Hu et al., 2020).

CELF1 and ELAVL1 proteins, in addition to their cytoplasmic roles, have been found directly interacting in the nucleus, where they cooperatively control the splicing of CD44 in HeLa cells (David et al., 2022). Namely, they promote the inclusion of exons v7-v10. Correlation analysis of the alternative splicing events of CD44 with expression levels of *CELF1* and *ELAVL1* based on RNA-Seq data from TCGA revealed that high expression of *CELF1* and/or *ELAVL1* is correlated with the inclusion of CD44 variable exons in eight tumor types.

In experiment combination, it has been shown that SRm160 (encoded by the *SRRM1* gene) is important for the inclusion of most of the endogenous CD44 variable exons in HeLa cells (Cheng and Sharp, 2023). The regulation of CD44 splicing by SRm160 occurs in a Ras-dependent manner. Reduction of SRm160 by siRNA transfection downregulated the endogenous levels of CD44 variant isoforms and correlated with a decrease in HeLa cell invasiveness. In immunoprecipitation assay an association of SRm160 with Sam68 has been revealed (Cheng and Sharp, 2023), which in turn also stimulated the formation of CD44 variant isoforms in a Ras-dependent manner (Matter et al., 2002; Cheng and Sharp, 2023). The patterns of CD44 variant exons’ inclusion in HeLa cells treated with Sam68 siRNA were like those treated with SRm160 siRNA. These results suggest that SRm160 with Sam68 may interact to regulate CD44 splicing.

The splicing activity of Sam68 is dependent on the type of a complex it is a part of (Huot et al., 2009). A large Sam68 complex (>1 MDa) is a ribonucleoprotein complex composed of ∼40 proteins. The treatment of HeLa cells by phorbol 12-myristate 13-acetate or epidermal growth factor induced the disassociation of Sam68 from this large complex and the appearance of Sam68 within the smaller complex. In human MCF-7 and BT-20 breast cancer cells Sam68 exists in equilibrium between a large and a small complex, whereas MDA-MB-231 cells harbors only the smaller Sam68 complex. The appearance of the small Sam68 complex in the cells correlated with the ability of Sam68 to promote the inclusion of exon v5 in the CD44 minigene system and cell migration (Huot et al., 2009). The existence of Sam68 in the form of a protein complex provides multiple opportunities for cell-type-specific regulation of its splicing activity. Thus, the interaction of Sam68 with SND1 in prostate cancer cells leads to a synergic effect with Sam68 on variant exon inclusion in *CD44* mRNA (Cappellari et al., 2013). It has been demonstrated that SND1 affected the recruitment of Sam68 and snRNPs on *CD44* pre-mRNA. These results, in combination with others provided by (Cappellari et al., 2013), suggest that SND1 acts as a bridge between RNA polymerase II (RNAPII) and Sam68 and has a crucial role in CD44 AS by favoring the recruitment of the spliceosome and the efficient splicing of the variant exons. Knockdown of SND1, or Sam68, reduced proliferation and migration of prostate cancer cells.

In prostate cancer PC3 cells, overexpression of RBM3 protein resulted in decreased expression of CD44 isoform 3 (CD44v8-v10) and an increased expression of CD44 isoform 4 (CD44s) (Zeng et al., 2013). Vice versa, decreasing the expression of RBM3 promoted the expression of CD44 isoform 3 and suppressed the expression of isoform 4. These results suggested that RBM3 promoted switching from CD44 isoform 3 to isoform 4. Such switching, in turn, attenuated CSC-like features of prostate cancer cells. This finding is confirmed by the fact that RBM3 overexpression in PC3 cells showed a significant reduction in tumor formation when cells were inoculated in nude mice.

The MBNL3 protein is a splicing regulator promoting embryonic stem cell differentiation (Han et al., 2013). Knockdown of MBNL3 in acute myeloid leukemia stem cells enhanced the expression of the CD44 isoform 3, which promoted stem cell maintenance (Holm et al., 2015).

Interestingly, in studies with MCF7 and HEK293T cells, it has been found that binding of the acetyltransferase p300 to the CD44 promoter region stimulated the inclusion of variant exons v5-v6 in *CD44* mRNA independently of RNAPII transcriptional elongation rate (Siam et al., 2019). The mechanism of AS regulation by p300 included an acetylation of splicing factors, leading to the exclusion of hnRNPM from *CD44* pre-mRNA and activation of Sam68.

U2AF2 knockdown and overexpression experiments revealed its positive regulatory role in the inclusion of variant exons and CD44 isoform 3 expression in melanoma cells (Zhang et al., 2016). It has been demonstrated that U2AF2 can bind to weak polypyrimidine tract in the 3′-splicing site to facilitate CD44 isoform 3 splicing. The U2AF2 activity could be inhibited by the CD82 tetraspanin protein by inducing U2AF2 ubiquitination. Knockdown of U2AF2 or CD44 isoform 3 significantly diminished the adhesion to E-selectin of Lu1205M melanoma cells and reduced the number of metastatic lesions. Of note, silencing of a set of other splicing factors in this study, Tra2β, SRp20, ESRP1, YB-1, SRm160, and Sam68, did not show any changes in the expression level of CD44 isoform 3 in melanoma cells (Zhang et al., 2016).

PCBP1 (alpha CP1 or hnRNPE1) has been characterized as a negative regulator of CD44 variants splicing in the human hepatoma cell line HepG2 (Zhang et al., 2010). An enforced expression of PCBP1 inhibited CD44 variant isoforms expression, including v3, v5, v6, v8, and v10 exons, while knockdown of endogenous PCBP1 induced CD44 variant isoforms splicing. The PCBP1 overexpression was accompanied by a decrease in invasive features of tumor cells; the knockdown accordingly promoted invasion.

Reduced expression of hnRNPL (the heterogeneous nuclear ribonucleoprotein L) promoted inclusion of only exon v10 in endogenous *CD44* mRNA in MDA-MB-231 cells (Loh et al., 2015). A similar result has been shown using the CD44 exon v10 minigene reporter system in MDA-MB-231 and HCT-116 cells. In addition, it has been revealed that hnRNPL directly interacts with the CA-rich sequence in the intron upstream of CD44 exon v10. This interaction inhibited the recruitment of U2AF2 on intron upstream exon v10 and prevented its splicing.

HNRNPLL, a paralog of HNRNPL, has been demonstrated as a negative regulator of invasion and metastasis of mouse colorectal cancer CMT93 cells, which may be caused in part by its negative regulation of splicing CD44 isoforms containing exon v6 (Sakuma et al., 2018). In human colon cancer SW480 cells, reduced level of HNRNPLL enhanced expression of the CD44 isoforms containing exons v3-v10 and cell invasion activity. Induction of EMT in SW480 cells led to transcriptional downregulation of HNRNPLL and upregulation of exon v6 inclusion in *CD44* mRNA.

In the study described by Muys et al. CD44 was the strongest alternatively spliced target of ZMAT3 in HCT116 cells (Muys et al., 2021). Silencing of ZMAT3 resulted in a higher abundance of CD44 variant isoform 1 and isoform 2, a concomitant reduction of the short standard CD44 isoform 4 and an increase in clonogenicity of HCT116 cells. ZMAT3 regulation of CD44 splicing may be related to its binding at pyrimidine-rich sequences of pre-mRNA introns, a crucial sequence element required for 3′ splice site definition. Most commonly, ZMAT3 binding sites consist of Us, with additional significant contribution of A/U-rich elements (AREs). Thus, ZMAT3 might compete with the other ARE-binding RBPs and splicing machinery for binding and interfere with properly recognizing 3′ splice sites (Muys et al., 2021).

In experiments with HeLa cells, A/C-rich elements (ACE) in CD44 exon v4 were recognized by the human YB-1 protein, encoding the *YBX1* gene and initially identified as a transcription factor (Stickeler et al., 2001). The YB-1 binding to the exonic ACE stimulated CD44 exons v4 and v5 inclusion in the final transcript.

## 5 Conclusion and prospects

Alternative splicing of *CD44* pre-mRNA and the essential role of CD44 isoforms in cancers are highlighted in this review. Many RBPs have been identified as regulators of CD44 isoform splicing, of which the most studied regulator is ESRP1 (Table 2, the extended version of the table see in Supplementary Table S1). RBPs typically exist as an important part of larger protein complexes that provide multiple opportunities for cell-type specific regulation of their splicing activity. Moreover, RBPs may counteract each other [e.g., as was shown for SRSF1/2 and NSrp70 (Kim Y. D. et al., 2011; Kim et al., 2016) or hnRNPM and AKAP8 (Hu et al., 2020)] and their expression ratio could be important [e.g., as it was shown for RBFOX2 and ESRP1 (Barriere et al., 2014; Meng et al., 2019)]. All of these necessitate further research into the role of RBPs mentioned in the review in each type of cancer and the identification of other possible regulators of CD44 alternative splicing. The importance of studying endogenous AS in cells is worth noting, since the model systems such as the CD44 minigene splicing reporter systems provide only limited information and do not fully reflect endogenous processes in cells.

It is also important to note that, according to sequencing data of full-length RNA transcripts from the FLIBase repository (Shi et al., 2023), CD44 splicing may be more complex than the inclusion or exclusion of cassette exons and may involve changes in the sequence of the exons themselves. How these types of CD44 isoforms are realized and what functional role they play remains to be studied. The fact that six isoforms of eight, confirmed per the NCBI database, are very low expressed and are not even included in the top 20 high-expressed ones (Supplementary Figures S1, S2), according to FLIBase, also deserves particular discussion.

Several studies demonstrated that CD44 is a potential therapeutic target among various malignant entities, e.g., triple-negative basal-like breast cancer, squamous cell carcinomas, and acute myelogenous leukemia (Yan et al., 2015; Xu et al., 2020; Elakad et al., 2022). However, the results of preclinical and clinical trials showed not only the safety and efficacy of existing anti-CD44 therapies but their limited success also [the detailed information were nicely summarized in (Xu et al., 2020; Primeaux et al., 2022; Weng et al., 2022)].

**FIGURE 5:**
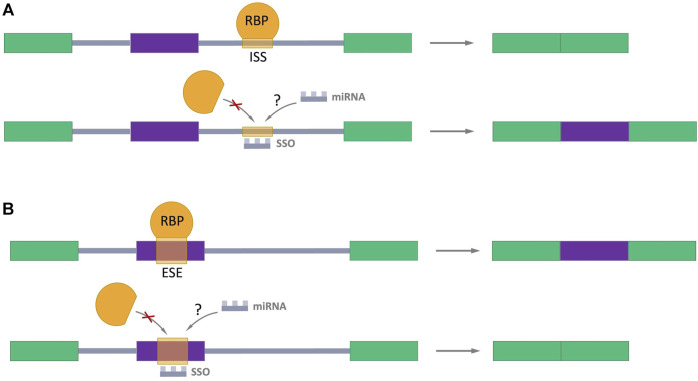
Splicing-switch oligonucleotides (SSOs) in AS regulation. **(A)** An SSO that binds to an intronic splicing silencer (ISS) and prevents binding of RNA-binding protein (RBP) which negatively regulates splicing (shown in orange), leading to exon inclusion. **(B)** An SSO that binds to an exonic splicing enhancer (ESE) and blocks the binding of RBP which promotes splicing (shown in orange), resulting in exon skipping. Exons are represented as color boxes, introns as gray lines.

Many studies have been devoted to exploring the possibility of splicing regulation through splicing-switch oligonucleotides (SSOs) which can specifically bind to splicing sites in the pre-mRNA in a complementary pairing manner, preventing RBPs binding and the normal assembly of spliceosome (Figure 5) (Hong, 2017; Du et al., 2021; Roy Burman et al., 2021; Zhang et al., 2021). Such oligonucleotides are analogs of the antisense oligonucleotides (ASOs), which the FDA has approved for the treatment of Duchenne muscular dystrophy (Lim et al., 2017) and spinal muscular atrophy (Corey, 2017). This approach provides a hope for perspective using SSOs as regulators of AS in cancer treatment. Interestingly, the endogenous prototypes of SSOs are miRNAs. It raises the important question: Could natural miRNAs be the regulators of AS in cells? The answer of this question definitively defines direction for future studies.

## Supplementary material

SUPPLEMENTARY FIGURE S1 Mean expression of CD44 isoforms across different cancers according to the FLYBase database.

SUPPLEMENTARY FIGURE S2 The mainly expressed CD44 splicing isoforms according to the FLIBase database.

## Abbreviations

AS, Alternative splicing; ASOs, Antisense Oligos; AREs, A/U-rich elements; ACE, A/C-rich elements; CRC, Colorectal cancer; CSC, Cancer stem cells; ESE, Exonic splicing enhancer; ISS, Intronic splicing silencer; iPSCs, Pluripotent stem cells; RBPs, RNA binding proteins; RNAPII, RNA polymerase II; RRM, RNA-recognition motif; siRNA, Small interfering RNA; shRNA, Small hairpin RNA; SSOs, Splicing-switch oligonucleotides.
