# Supplementary material for: RNA-binding proteins regulating the CD44 alternative splicing
Source: Front Mol Biosci. 2023 Dec 1;10:1326148. doi: 10.3389/fmolb.2023.1326148 (PMC10722200; doi:10.3389/fmolb.2023.1326148)
Supplement: Supplementary file 1 [file Table1.docx]

Supplementary Material

# Supplementary Table 1 RNA-binding proteins regulating CD44 variant exon splicing

| **Protein** | **Effect** | **Isoform changes** | **Other outcomes** | **Cancer type, cell line** | **Reference** |
| --- | --- | --- | --- | --- | --- |
| ESRP1 | Ectopic expression of FLAG-tagged ESRP1 | Switching from primarily CD44 isoform 4 expression to isoforms containing variant exons (including CD44 isoform 3 and CD44v6-v10). |  | Human breast cancer cell line MDA-MB-231 | (Warzecha et al., 2009a) |
| ESRP1 | Ectopic expression of mouse Esrp1 | Switching CD44 isoform 4 to variant isoforms. |  | Human breast cancer cell line MDA-MB-231 | (Warzecha et al., 2009b) |
| ESRP1 | Stable shRNA-mediated knockdown | Switching CD44 variant isoforms including isoform 3 to isoform 4. | - The incidence and extent of lung metastasis was reduced (tumor cells were orthotopically injected into mouse mammary glands). | Mouse breast cancer cell line 4T1 | (Yae et al., 2012) |
| ESRP1 | Knockdown of ESRP1 by two different shRNAs | Shift in expression from CD44 variant isoforms to isoform 4. | - The isoform switch to CD44 isoform 4 was required for the formation of breast tumors in mice. - CD44 isoform 4 activated Akt signaling. | Human mammary epithelial cell line HMLE | (Brown et al., 2011) |
| ESRP1 | ESRP1 overexpression | Absence of switching CD44 variant isoforms to isoform 4 during the course of Snail-induced EMT. |  | Human mammary epithelial HMLE/Snail-ER cell line | (Reinke et al., 2012) |
| ESRP1 | shRNA-mediated knockdown | Accelerated isoform switching from CD44 variant isoforms to isoform 4 in response to TGFβ treatment. | - Enhanced mammosphere-forming ability in response to TGFβ treatment. - Silencing CD44 in the ESRP1-depleted cells abrogated mammosphere formation. - CD44 isoform 4 activates the PDGFRβ/Stat3 cascade to promote CSC traits. | Human mammary epithelial cell line HMLE | (Zhang et al., 2019) |
| ESRP1 | Ectopic expression of ESRP1 | Reduced expression of CD44 isoform 4. | Great inhibition of the CSC gene signature. | TGFβ-treated HMLE cells, where endogenous ESRP1 was low | (Zhang et al., 2019) |
| ESRP1 | A transient knockdown | Shift in expression from CD44 variant isoforms containing exon v6 to CD44 isoform 4 without affecting total CD44 level. |  | Human breast cancer MCF7 and pancreatic adenocarcinoma BxPC-3 cell lines. | (Preca et al., 2015) |
| ESRP1 | Overexpression of ESRP1 | Switching from CD44 isoform 4 to CD44 variant isoforms containing exon v6. | Decreased ZEB1 levels | Human undifferentiated, mesenchymal breast cancer MDA-MB231 and pancreatic ductal adenocarcinoma Panc-1 cell lines | (Preca et al., 2015) |
| ESRP1 | Stable knockdown using sh-ESRP1 lentivirus vector | Switching from CD44 variant isoforms containing exon v7 to isoform 4 without change in total amount of CD44. | Increased migration and invasion. | Human epithelial ovarian cancer cell lines HO8910 | (Chen et al., 2017) |
| ESRP1 | Stable ectopic overexpression of ESRP1 | Switching from CD44 isoform 4 to variant isoforms. | Overall switching from mesenchymal to epithelial phenotype of cells. | Human ovarian cancer cell line SKOV3 | (Jeong et al., 2017) |
| ESRP1 | ESRP1 silencing by transfection with dicer-substrate siRNA (two siRNAs) | Upregulation of the standard CD44 isoform 4 and downregulation of the CD44 variant isoforms. |  | Human colorectal cancer cell line HCT-116 | (Vadlamudi and Kang, 2022) |
| ESRP1 | Overexpression ESRP1 variants v1, v4, or v5 | Conversion from CD44v9-v10 to CD44v7-v10. |  | Human fully differentiated human foreskin fibroblasts | (Kim et al., 2018) |
| ESRP1 | Stable knockdown mediated by shRNA lentiviral transduction. | Downregulation of CD44v7-v10 expression and upregulation of CD44v9-v10. |  | Undifferentiated H9 human embryonic stem cell | (Kim et al., 2018) |
| ESRP1 | siRNA-mediated knockdown | Switching from the CD44 variant isoforms to the CD44 isoform 4. | Enhanced cell motility. | Human head and neck squamous cell carcinoma cell lines SAS and HSC4 | (Ishii et al., 2014) |
| ESRP1 | siRNA-mediated knockdown | The decreased expression of CD44 isoforms containing exon v6. | - Significantly reduced migration of cells under HGF treatment. | Human cell lines MB and LH derived from melanoma lymph node metastases. | (Marzese et al., 2015) |
| ESRP1 | Ectopic expression of FLAG-tagged ESRP1 | Significant downregulation of CD44 overall expression. |  | Mesenchymal melanoma cell line MDA-MB-435 (Prasad and Gopalan, 2015) | (Warzecha et al., 2009a) |
| ESRP1 | Cotransfection of CD44 variable exon 5 minigene with ESRP1. | Promoted exon v5 inclusion. |  | Human embryonic kidney cell line HEK293FT. | (Harvey et al., 2018) |
| ESRP1 | siRNA-mediated knockdown | No effects on CD44 transcripts’ levels. |  | Human melanoma cell line Lu1205M. | (Zhang et al., 2016) |
| ESRP1 and ESRP2 | Simultaneous depletion by transfection with siRNAs against *ESRP1* and *ESRP2*. | Significant decrease in the inclusion of CD44 variant exons (mainly exons v8–v10) and increase in the standard CD44 isoform 4. | - Increased expression of the mesenchymal isoforms of p120-catenin and FGFR2. - Silencing epithelial-specific isoform of ENAH. | Normal human prostatic epithelial cell line PNT2 | (Warzecha et al., 2009a) |
| ESRP1 and ESRP2 | Loss of ESRPs expression due to the induction of an EMT by the transcription factor Twist. | Switching from CD44 isoform 4 to variant isoforms. |  | Human mammary epithelial cell line HMLE | (Warzecha et al., 2009a) |
| ESRP2 | siRNA-mediated knockdown | No effects on CD44 isoform expression. | Enhanced cell motility | Human head and neck squamous cell carcinoma cell lines SAS and HSC4 | (Ishii et al., 2014) |
| RBFOX2 | Bioinformatic prediction | Negative regulator of variant exon inclusion in *CD44* mRNA. | - EMT upregulates the expression of RBFOX2. - During an EMT, RBFOX2 regulates splicing shifts from epithelial to mesenchymal-specific events, leading to a higher degree of tissue invasiveness. | Colon adenocarcinoma samples of patients | (Danan-Gotthold et al., 2015) |
| RBFOX2 | siRNA-mediated knockdown | No effects on inclusion of exons v8-v10 in *CD44* mRNA. |  | Mouse non-transformed mammary epithelial cell line NMuMG and epithelial murine breast cancer cell line PY2T | (Braeutigam et al., 2013) |
| RBFOX2 and ESRP1 | Upregulation of long transcript variant of RBFOX2 and downregulation of short variant of RBFOX2 and ESRP1 in response to ectopic expression of WNT5A | Downregulated inclusion of exons v4-v6 (but not v9) in CD44 mRNA. | - Reduced cell migration. - less lung metastasis. | Mouse breast cancer cell line 4T1 | (Jiang et al., 2013) |
| QKI | Bioinformatic prediction | A potential negative regulator of CD44 isoform 3 formation. |  | Tumor samples of patients with colorectal cancer. | (Novosad, 2023) |
| PTBP1 | siRNA-mediated knockdown | Decreased expression of CD44 isoforms containing exon v6. | - M16 cells showed a significant decrease in cell migration. - BD cells showed a significant increase in cell migration. | Human melanoma brain metastases’ cell lines BD and M16. | (Marzese et al., 2015) |
| PTBP1 | Co-transfection of CD44 exon v8 minigene splicing reporter construct and PTBP1 cDNA. | Increased exon v8 inclusion. |  | Human embryonic kidney cell line HEK293FT. | (Hu et al., 2020) |
| NONO | Simultaneous transfection with CD44 minigene reporter construct containing exons v4-v5 and NONO vector. | Decreased ratio of skipping to inclusion of exons v4-v5 in CD44 minigene reporter system. |  | HEK293T cells. | (Liu et al., 2011) |
| NONO | Overexpression by transfection of cells with NONO vector. | No effects on CD44 transcripts levels. |  | Human cervical carcinoma HeLa cell line. | (Zhao et al., 2022) |
| NONO | Simultaneous transfection with CD44 minigene reporter construct containing exons v4-v5 and NONO vector. | Decreased ratio of skipping to inclusion of exons v4-v5 in CD44 minigene reporter system. |  | Human cervical carcinoma HeLa cells. | (Zhao et al., 2022) |
| SFPQ | Simultaneous transfection with CD44 minigene reporter construct containing exons v4-v5 and SFPQ vector. | Increased ratio of skipping to inclusion of exons v4-v5 in CD44 minigene reporter system. |  | Human embryonic kidney HEK293T cells. | (Liu et al., 2011) |
| SFPQ | Overexpression by transfection of cells with SFPQ vector. | No effects on CD44 transcripts’ levels. |  | Human cervical carcinoma HeLa cell line. | (Zhao et al., 2022) |
| SFPQ | Simultaneous transfection with CD44 minigene reporter construct containing exons v4-v5 and SFPQ vector. | Significant increase of variant exon inclusion and decrease of CD44 isoform 4 level. |  | Human cervical carcinoma HeLa cell line. | (Zhao et al., 2022) |
| SFPQ | Stable shRNA-mediated knockdown. | Reduced expression of CD44 isoforms containing exon v6. | - Inhibition of cell stemness. - Inhibition of cell proliferation in vitro. - Reduction of metastasis in mice. | Human lung cancer mesenchymal stem cells isolated from lung tissue biopsies. | (Yang et al., 2022) |
| AGGF1 | Overexpression by transfection of cells with AGGF1 vector. | Enhanced inclusion of exons v4 and v5 (but not v8-v10 or v10) in CD44 mRNA and decreased level of CD44 isoform 4 |  | HeLa cells and HeLa cells cotransfected with CD44 minigene reporter and AGGF1 vector. | (Zhao et al., 2022) |
| AGGF1 | Co-overexpression of AGGF1 with NONO or SFPQ, or DHX15. | Decreased ratio of skipping to inclusion of exons v4-v5 in CD44 minigene reporter system. |  | HeLa cells simultaneously transfected with CD44 minigene reporter and NONO vector or SFPQ vector, or DHX15 vector. | (Zhao et al., 2022) |
| DHX15 | Simultaneous transfection with CD44 minigene reporter construct containing exons v4-v5 and DHX15 vector. | Decreased ratio of skipping to inclusion of exons v4-v5 in CD44 minigene. |  | Human cervical carcinoma HeLa cell line. | (Zhao et al., 2022) |
| hnRNPM | shRNA-mediated knockdown. | Completely abolished TGFβ-induced CD44 isoform switching from CD44 variant isoforms to isoform 4. | - Inhibition of TGFβ-induced EMT. - Reduction of metastasis in mice. - Forced expression of the CD44 isoform 4 rescued the impaired EMT phenotype that occurred in the hnRNPM-depleted cells and permited metastasis in mice. | Human mammary epithelial cell line HMLE, murine T4 breast cancer cell, and human LM2 cells (MDA-MB-231 derivatives cells). | (Xu et al., 2014) |
| hnRNPM | Co-transfection hnRNPM and CD44v8 minigene splicing reporter construct. | Increased exon v8 skipping in a hnRNPM dose-dependent manner. | Mutating the I-8 GU motifs in the splicing minigene construct or swapping the 200-nt intronic region, including I-8, with a random 200-nt sequence almost completely abolished the ability of hnRNPM to promote exon skipping. | Human embryonic kidney cell line HEK293. | (Xu et al., 2014) |
| hnRNPM | shRNA-mediated knockdown (two two shRNAs were used) | Significantly decrease in CD44 isoform 4 with a concomitant increase in CD44v5/6 and v8/9 mRNA. |  | TGFβ-inducted mesenchymal human breast cancer cell line MCF10A (Mes10A). | (Xu et al., 2014) |
| hnRNPM | Co-transfection hnRNPM and CD44 variable exon v8 minigene splicing reporter construct. | - No effects on CD44 exon v8 skipping. - ESRP1-knockdown in HCT116 cells restores hnRNPM’s ability to promote exon skipping. |  | Human colorectal cancer cell line HCT-116. | (Xu et al., 2014) |
| hnRNPM | shRNA-mediated knockdown (two different shRNAs were used) | Reversed the mutant MORC2-induced CD44 splicing switch (decreased CD44 isoform 4 with a concomitant increase in CD44 containing exons v5-6) | - Reduced binding of mutant MORC2 to CD44 pre-mRNA. - Reduced EMT. - Impairing the migratory, invasive, and lung metastatic potential of mutant MORC2-expressing cells. | Normal human breast epithelial cell line MCF10A stably expressing M276I-mutant form of MORC2. | (Zhang et al., 2018) |
| hnRNPM | Overexpression by transfection with a hnRNPM expression plasmid. | Decreased expression in CD44 isoforms containing exon v6 and increased expression in CD44 isoform 4 with a slight change of total level of CD44 transcripts | Enhanced invasiveness of breast cancer cells. | Human breast cancer cell line MCF-7. | (Sun et al., 2017) |
| hnRNPM | Co-transfection of CD44 variant exon v5 minigene splicing reporter construct and hnRNPM plasmid. | Increased exon v5 skipping. |  | Human embryonic kidney cell line HEK293FT. | (Harvey et al., 2018) |
| hnRNPM | Co-transfection of CD44 minigene splicing reporter construct containing exon v8 or v5 and hnRNPM cDNA. | Increased exon v8 or v5 skipping, respectively. | - The presence of AKAP8 dampened the effect of hnRNPM on promoting CD44 exon v8 exclusion. - AKAP8 silencing led to a more drastic effect of hnRNPM on exon skipping in both CD44v8 and CD44v5 minigenes. | Human embryonic kidney cell line HEK293FT. | (Hu et al., 2020) |
| hnRNPF | Co-transfection of CD44 minigene splicing reporter construct containing exon v8 and hnRNPF cDNA. | Increased exon v8 inclusion. |  | Human embryonic kidney cell line HEK293FT. | (Hu et al., 2020) |
| hnRNPR | Co-transfection of CD44 minigene splicing reporter construct containing exon v8 and hnRNPR cDNA. | Increased exon v8 skipping. |  | Human embryonic kidney cell line HEK293FT. | (Hu et al., 2020) |
| AKAP8 | Co-transfection of CD44 minigene splicing reporter construct containing exon v8 and AKAP8 cDNA. | Increased exon v8 inclusion in AKAP8 dose-dependent manner. | SiRNA-mediated silencing of hnRNPM showed a moderate but not significant increase of the AKAP8’s splicing activity | Human embryonic kidney cell line HEK293FT. | (Hu et al., 2020) |
| RBM10 | Co-transfection of CD44 minigene splicing reporter construct containing exon v8 and RBM10 cDNA. | Increased exon v8 skipping. |  | Human embryonic kidney cell line HEK293FT. | (Hu et al., 2020) |
| RBMX | Co-transfection of CD44 minigene splicing reporter construct containing exon v8 and RBMX cDNA. | Increased exon v8 skipping. |  | Human embryonic kidney cell line HEK293FT. | (Hu et al., 2020) |
| CELF1 | siRNA-mediated knockdown. | Reduced inclusion of variable exons v7 to v10 into mature *CD44* mRNAs. |  | Human cervical carcinoma HeLa cell line. | (David et al., 2022) |
| ELAVL1 | siRNA-mediated knockdown. | Reduced inclusion of variable exons v7 to v10 into mature *CD44* mRNAs. |  | Human cervical carcinoma HeLa cell line. | (David et al., 2022) |
| CELF1 and  ELAVL1 | Simultaneous siRNA-mediated knockdown of both proteins. | Depleting both proteins reduced incorporation of variable exons v7 to v10 into mature *CD44* mRNAs even more than each protein alone. |  | Human cervical carcinoma HeLa cell line. | (David et al., 2022) |
| SRSF1 | siRNA-mediated knockdown. | Significantly decrease in CD44 isoform 3 (but not in isoforms containing exon v6 or exons v6-v10) with a concomitant increase in CD44 isoform 4 mRNA level. |  | Human gastric carcinoma cell line MGC-803. | (Peng et al., 2019) |
| SRSF1 | Overexpression by transfection with a SRSF1 expression plasmid. | Switching from CD44 isoform 4 to isoform 3. |  | Human gastric carcinoma cell line MGC-803. | (Peng et al., 2019) |
| SRSF1 | Overexpression by transfection with a SRSF1 expression plasmid. | Increased exon v6 skipping in CD44 minigene splicing reporter system. |  | Human breast cancer MCF7 stable cell line expressing the pFlare-V6 plasmid (a kind of CD44 minigene reporter system containing CD44 variant exon v6). | (Loh et al., 2016) |
| SRSF1 | shRNA-mediated knockdown | Decreased expression of CD44v6-v10 and CD44v6,v8-v10 isoforms. |  | Human breast cancer MCF7 cell line. | (Loh et al., 2016) |
| SRSF1 | Co-transfection of CD44 exon v5 minigene splicing reporter construct with SRSF1 plasmid | Increased exon v5 skipping. |  | Human embryonic kidney cell line HEK293T. | (Kim et al., 2016) |
| SRSF2 | Overexpression by transfection with a SRSF2 expression plasmid. | Increased exon v6 skipping in CD44 minigene splicing reporter. |  | Human breast cancer MCF7 stable cell line expressing the pFlare-V6 plasmid (a kind of CD44 minigene splicing reporter system containing CD44 variant exon v6). | (Loh et al., 2014) |
| SRSF2 | shRNA-mediated knockdown | Decreased expression of CD44v6 isoform but increased expression of CD44v6-v10 and CD44v6,v8-v10 isoforms. |  | Human breast cancer MCF7 cell line. | (Loh et al., 2016) |
| SRSF2 | Co-transfection of CD44 exon v5 minigene splicing reporter construct with SRSF2 plasmid | Increased exon v5 skipping. |  | Human embryonic kidney cell line HEK293T. | (Kim et al., 2016) |
| SRSF3 | Overexpression by transfection with a SRSF3 expression plasmid. | No effects on exon v6 splicing. |  | Human breast cancer MCF7 stable cell line expressing the pFlare-V6 plasmid (a kind of CD44 minigene reporter system containing CD44 variant exon v6) | (Loh et al., 2016) |
| SRSF3 | shRNA-mediated knockdown | Decreased expression of CD44v6-v10 and CD44v6,v8-v10 isoforms. |  | Human breast cancer MCF7 cell line | (Loh et al., 2016) |
| SRSF3 | Stable shRNA-mediated knockdown (two different shRNA were used). | Decreased expression of CD44 variant isoforms but increased expression the CD44 isoform 4. | The reduction of CD44 variant isoform expression due to SRSF3 silencing could be partially rescued through elevation of TDP43. | Human triple-negative breast cancer cell lines HCC1806 and MDA-MB-231. | (Guo et al., 2022). |
| SRSF3 | Stable overexpression. | Increased expression in CD44 variant isoforms, while total CD44 transcription did not change |  | Human triple-negative breast cancer cell lines HCC1806 and MDA-MB-231. | (Guo et al., 2022). |
| SRSF4 | Overexpression by transfection with a SRSF4 expression plasmid. | No effects on exon v6 splicing. |  | MCF7 stable cell line expressing the pFlare-V6 plasmid (a kind of CD44 minigene reporter system containing CD44 variant exon v6). | (Loh et al., 2016) |
| SRSF4 | shRNA-mediated knockdown | No effects on CD44 transcripts’ levels. |  | Human breast cancer MCF7 cell line. | (Loh et al., 2016) |
| SRSF6 | Overexpression by transfection with a SRSF6 expression plasmid. | Increased exon v6 skipping. |  | MCF7 stable cell line expressing the pFlare-V6 plasmid (a kind of CD44 minigene reporter system containing CD44 variant exon v6). | (Loh et al., 2016) |
| SRSF9 | Overexpression by transfection with SRSF9 expression plasmid. | Increased exon v6 skipping. |  | MCF7 stable cell line expressing the pFlare-V6 plasmid (a kind of CD44 minigene reporter system containing CD44 variant exon v6). | (Loh et al., 2016) |
| SRSF9 | shRNA-mediated knockdown | No effects on CD44 transcripts’ levels. |  | Human breast cancer MCF7 cell line. | (Loh et al., 2016) |
| SRSF9 | Co-transfection of CD44 exon v10 minigene splicing reporter construct with SRSF9 plasmid. | Reduced CD44 exon v10 inclusion. |  | Human embryonic kidney cell line HEK293T and colorectal cancer cell line HCT116. | (Oh et al., 2020) |
| SRSF9 | shRNA-mediated knockdown | No effect on endogenous CD44 exon v10 splicing |  | Human embryonic kidney cell line HEK293T and colorectal cancer cell line HCT116. | (Oh et al., 2020) |
| Tra2β | Co-transfection of CD44 exon v10 minigene splicing reporter construct with Tra2β expression plasmid. | Increased CD44 exon v10 inclusion. |  | Human embryonic kidney cell line HEK293T and colorectal cancer cell line HCT116. | (Oh et al., 2020) |
| Tra2β | shRNA-mediated knockdown | No effect on endogenous CD44 exon v10 splicing |  | Human embryonic kidney cell line HEK293T and colorectal cancer cell line HCT116. | (Oh et al., 2020) |
| Tra2β | siRNA-mediated knockdown | No effects on CD44 transcripts’ levels. |  | Human melanoma cell line Lu1205M. | (Zhang et al., 2016) |
| NSrp70 | Co-transfection of CD44 exon v5 minigene splicing reporter construct with NSrp70 expression plasmid. | Increased CD44 exon v5 inclusion. | - NSrp70 counteracts SRSF1- and SRSF2-induced CD44 Exon v5 Exclusion | Human embryonic kidney cell line HEK293T. | (Kim et al., 2011, 2016) |
| SRm160 | siRNA-mediated knockdown | Decreased expression of CD44 variant isoforms. | Decrease in HeLa cell invasiveness. | Human cervical carcinoma HeLa cell line. | (Cheng and Sharp, 2023) |
| SRm160 | siRNA-mediated knockdown | No effects on CD44 transcripts’ levels. |  | Human melanoma cell line Lu1205M. | (Zhang et al., 2016) |
| Sam68 | siRNA-mediated knockdown | Decreased expression of CD44 variant isoforms. |  | Human cervical carcinoma HeLa cell line. | (Cheng and Sharp, 2023) |
| Sam68 | Co-transfection of CD44 exon v5 minigene splicing reporter construct with Sam68 expressing plasmid. | Stimulation of CD44 exon v5 inclusion after treatment with phorbol ester. |  | Mouse EL4 T-lymphoma cells. | (Matter et al., 2002) |
| Sam68 | Co-transfection of CD44 exon v5 minigene splicing reporter construct with Sam68 expressing plasmid. | Stimulation of CD44 exon v5 inclusion. | Simultaneous overexpression of SND1 led to a synergic effect with Sam68 on variant exon inclusion. | Human embryonic kidney cell line HEK293T. | (Cappellari et al., 2013) |
| Sam68 | Stable knockdown for SAM68. | Decreased inclusion of variable exons v4, v5, v7, v8, v9, v10 in *CD44* mRNA. The exons v4, v5 and v7 were more affected by SAM68 depletion. | Reduced proliferation and migration of prostate cancer cells. | Human prostate adenocarcinoma cell line PC3. | (Cappellari et al., 2013) |
| Sam68 | siRNA-mediated knockdown | No effects on CD44 transcripts’ levels. |  | Human melanoma cell line Lu1205M. | (Zhang et al., 2016) |
| RBM3 | Overexpression | Decreased expression of CD44 isoform 3 and increased expression of CD44 isoform 4. | - Attenuated CSC features of prostate cancer cells. - Significant reduction in tumor formation when cells were inoculated in nude mice. | Human prostate adenocarcinoma cell line PC3. | (Zeng et al., 2013) |
| RBM3 | siRNA-mediated knockdown | Increased expression of CD44 isoform 3 and decreased expression of CD44 isoform 4. |  | Human prostate adenocarcinoma cell line PC3. | (Zeng et al., 2013) |
| MBNL3 | Stable knockdown for MBNL3. | Upregulation of a CD44 splice isoform 3. | Activation of a pluripotency network. | Human acute myeloid leukemia stem cells | (Holm et al., 2015) |
| U2AF2 | siRNA- and shRNA-mediated knockdown | Significantly increased CD44 isoform 4 and attenuated CD44 isoform 3 expression levels. | - Diminished the adhesion probability of Lu1205M cells - Reduced the number of metastatic lesions. | Human melanoma cell line Lu1205M. | (Zhang et al., 2016) |
| U2AF2 | Overexpression of shRNA-resistant U2AF2 in U2AF2-silenced Lu1205M cells. | Rescued CD44 isoform 3 expression. |  | Human melanoma U2AF2-silenced Lu1205M cells. | (Zhang et al., 2016) |
| U2AF2 | Overexpression by transfection with U2AF2 expression plasmid. | Overexpression of U2AF2 triggered CD44 isoform 3 expression at both mRNA and protein levels |  | Human melanoma cell line SK-Mel-25. | (Zhang et al., 2016) |
| YB-1 | siRNA-mediated knockdown | No effects on CD44 transcripts’ levels. |  | Human melanoma cell line Lu1205M. | (Zhang et al., 2016) |
| SRp20 | siRNA-mediated knockdown | No effects on CD44 transcripts’ levels. |  | Human melanoma cell line Lu1205M. | (Zhang et al., 2016) |
| PCBP1 | Overexpression by transfection with PCBP1 expression plasmid. | Decreased expression of CD44 variant isoforms including v3, v5, v6, v8, and v10 exons.  No effect on exon v9 inclusion, and CD44 isoform 4 expression. | Decreased cell invasion. | Human hepatoma cell line HepG2. | (Zhang et al., 2010) |
| PCBP1 | siRNA-mediated knockdown | Increased expression of CD44 variant isoforms including v3, v5, v6, v8, and v10 exons. | Increased cell invasion. | Human hepatoma cell line HepG2. | (Zhang et al., 2010) |
| hnRNPL | shRNA-mediated knockdown | - Increased inclusion of only exon v10 in endogenous *CD44* mRNA. - A slight decreased exon v10 skipping in CD44 minigene system. - Overexpression of hnRNPL in the hnRNPL knocked-down cells restored exon v10 skipping in CD44 minigene system. |  | Human breast cancer cell line MDA-MB-231 and colorectal cancer cell line HCT-116. | (Loh et al., 2015) |
| hnRNPL | Co-transfection of CD44 exon v10 minigene splicing reporter construct with hnRNPL expressing plasmid. | Significantly increased exon v10 skipping in CD44 minigene system. |  | Human breast cancer cell line MDA-MB-231. | (Loh et al., 2015) |
| hnRNPLL | shRNA-mediated knockdown | Increased expression of CD44 isoforms containing exons v3-v10 and | Increased invasion activity of human colon cancer cells. | human colon cancer SW480 cell line. | (Sakuma et al., 2018) |
| hnRNPLL | shRNA-mediated knockdown | Increased expression of CD44 isoforms containing exon v6. | Significantly more metastatic nodules. | Mouse colorectal cancer CMT93 cell line. | (Sakuma et al., 2018) |
| ZMAT3 | shRNA-mediated knockdown. | Increased expression of CD44 variant isoforms 1 and 2, a concomitant reduction of standard CD44 isoform 4. | Increase in clonogenicity of tumor cells. | Human colorectal cancer cell line HCT116. | (Muys et al., 2021) |
| YB-1 | Co-transfection of CD44 minigene splicing reporter construct containing exons v4 and v5 with YB-1 expressing plasmid. | Increased the inclusion of CD44 exons v4 and v5. |  | Human cervical carcinoma HeLa cell line. | (Stickeler et al., 2001) |
| TDP43 | Stable shRNA-mediated knockdown (two different shRNA were used). | Decreased inclusion of variant exons in *CD44* mRNA (all variant exons in HCC1806 cells, and v10; exons v8, v9, and v10 in MDA-MB-231). | Reduced stemness features of breast cancer stem cells. | Human triple-negative breast cancer cell lines HCC1806 and MDA-MB-231. | (Guo et al., 2022). |
| TDP43 | Stable overexpression. | Increased inclusion of variant exons in *CD44* mRNA, especially exons v8, v9, and v10. |  | Human triple-negative breast cancer cell lines HCC1806 and MDA-MB-231. | (Guo et al., 2022). |
