# Supplementary figures and images for: RNA-binding proteins regulating the CD44 alternative splicing
Source: Front Mol Biosci. 2023 Dec 1;10:1326148. doi: 10.3389/fmolb.2023.1326148 (PMC10722200; doi:10.3389/fmolb.2023.1326148)

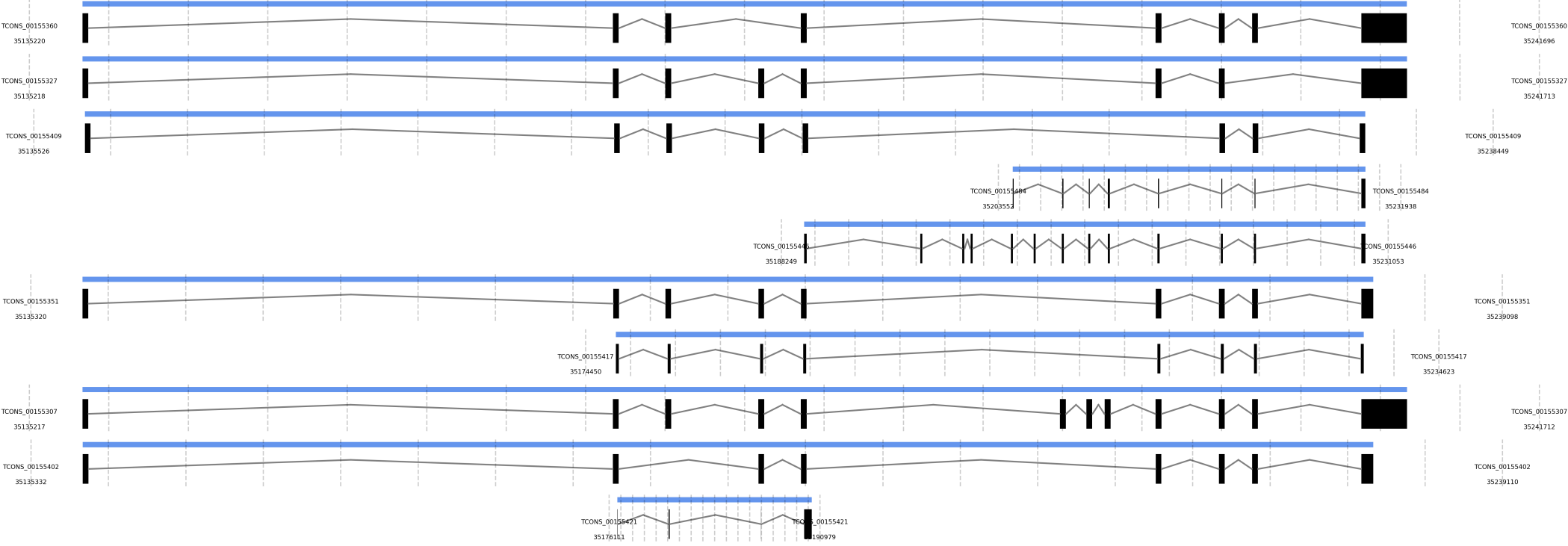

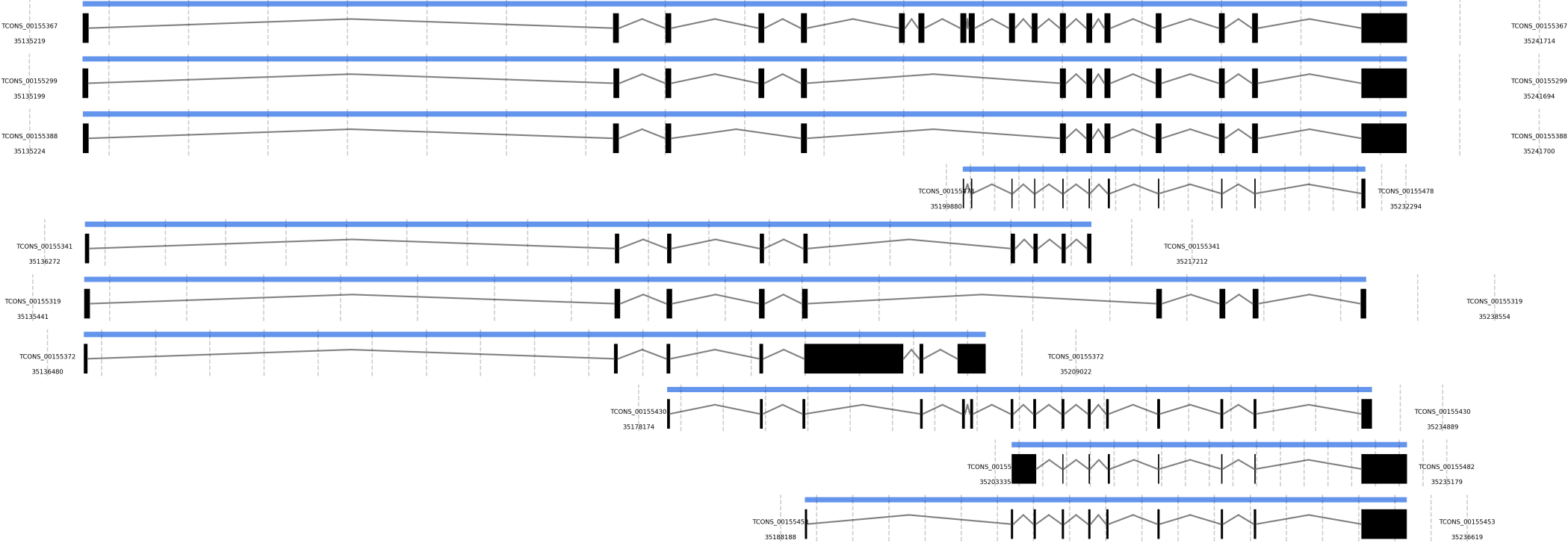

Supplement: Supplementary file 2 [file DataSheet1.PDF]

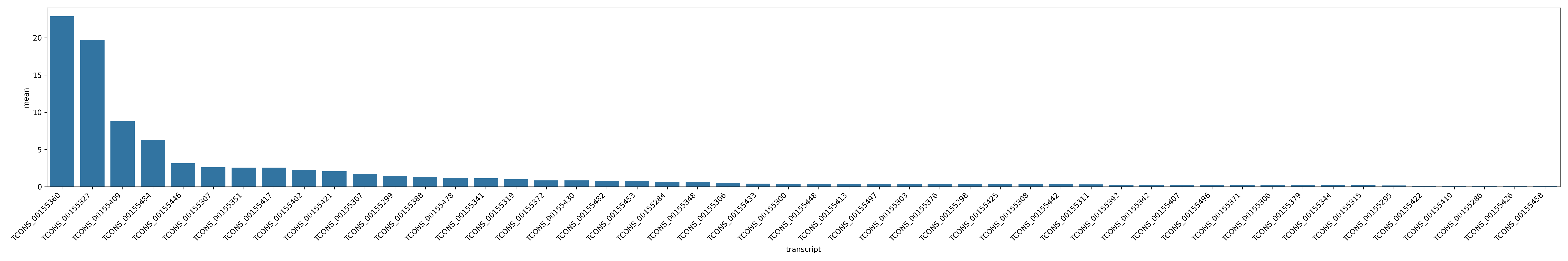

Supplement: Supplementary file 3 [file Image1.PNG]
